# Exploring Antibacterial Activity and Bacterial-Mediated Allotropic Transition of Differentially Coated Selenium Nanoparticles

**DOI:** 10.1021/acsami.3c05100

**Published:** 2023-06-09

**Authors:** Miguel A. Ruiz-Fresneda, Sebastian Schaefer, René Hübner, Karim Fahmy, Mohamed L. Merroun

**Affiliations:** † Department of Microbiology, University of Granada, Campus Fuentenueva, 18071 Granada, Spain; ‡ Institute of Resource Ecology, Helmholtz-Zentrum Dresden-Rossendorf, Bautzner Landstraße 400, 01328 Dresden, Germany; § Institute of Ion Beam Physics and Materials Research, Helmholtz-Zentrum Dresden-Rossendorf, Bautzner Landstraße 400, 01328 Dresden, Germany

**Keywords:** selenium, nanoparticles, antibiotic, bioremediation, applications

## Abstract

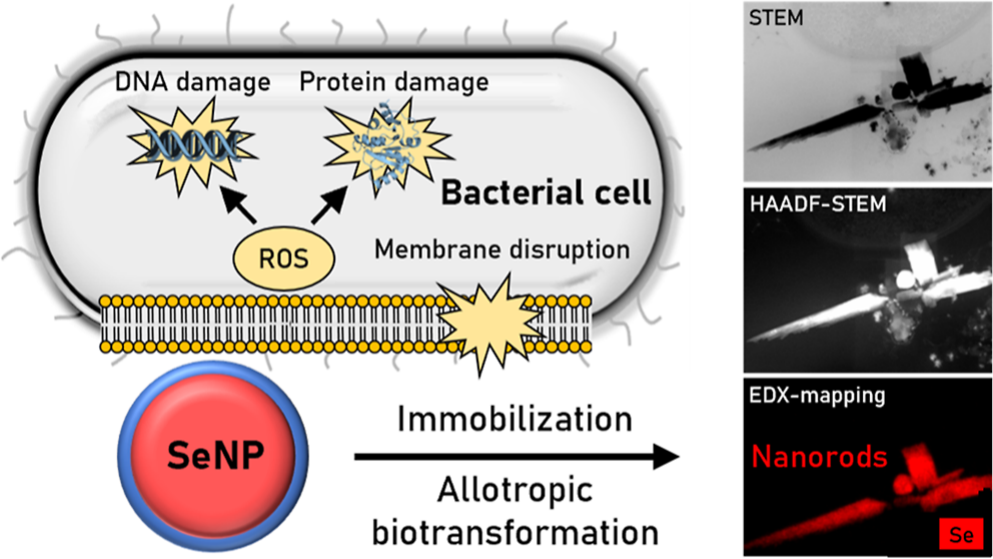

The use of metal nanoparticles (NPs) as antimicrobial agents has become a promising alternative to the problem of antibiotic-resistant bacteria and other applications. Silver nanoparticles (AgNPs) are well-known as one of the most universal biocide compounds. However, selenium nanoparticles (SeNPs) recently gained more attention as effective antimicrobial agents. This study aims to investigate the antibacterial activity of SeNPs with different surface coatings (BSA-coated, chitosan-coated, and undefined coating) on the Gram-negative *Stenotrophomonas bentonitica* and the Gram-positive *Lysinibacillus sphaericus* in comparison to AgNPs. The tested NPs had similar properties, including shape (spheres), structure (amorphous), and size (50–90 nm), but differed in their surface charge. Chitosan SeNPs exhibited a positive surface charge, while the remaining NPs assayed had a negative surface charge. We have found that cell growth and viability of both bacteria were negatively affected in the presence of the NPs, as indicated by microcalorimetry and flow cytometry. Specifically, undefined coating SeNPs displayed the highest percentage values of dead cells for both bacteria (85–91%). An increase in reactive oxygen species (ROS) production was also detected. Chitosan-coated and undefined SeNPs caused the highest amount of ROS (299.7 and 289% over untreated controls) for *S. bentonitica* and *L. sphaericus*, respectively. Based on DNA degradation levels, undefined-SeNPs were found to be the most hazardous, causing nearly 80% DNA degradation. Finally, electron microscopy revealed the ability of the cells to transform the different SeNP types (amorphous) to crystalline SeNPs (trigonal/monoclinical Se), which could have environmentally positive implications for bioremediation purposes and provide a novel green method for the formation of crystalline SeNPs. The results obtained herein demonstrate the promising potential of SeNPs for their use in medicine as antimicrobial agents, and we propose *S. bentonitica* and *L. sphaericus* as candidates for new bioremediation strategies and NP synthesis with potential applications in many fields.

## 1 Introduction

Nanoparticles (NPs) are materials with dimensions between 1 and 100 nm for at least one of their cross-sectional diameters.^1^ Due to the increased surface-to-volume ratio compared to bulk materials, optical, physical, and chemical properties of NPs can be significantly altered, thereby, enabling unique applications in health and industry.^2,3^ In this respect, metal NPs have previously been reported to exhibit antimicrobial activity.^4,5^ Apart from titanium oxide, copper oxide, or zinc oxide, silver NPs (AgNPs) display the most investigated antimicrobial particles and are of growing interest for applications in medicine.^4^

To achieve antimicrobial activity, physical contact of NPs with the net negatively charged cell membrane is required. Two major modes of interactions are proposed for bacteria, cellular uptake and adsorption based on electrostatic or van der Waals forces. The latter attractive forces are supposed to cause adsorption of NPs to the bacterial membrane, which are facilitated by a positive charge of NPs.^6^ Apart from surface properties, the size of NPs is crucial for the penetration and interactions within the bacterial cell and hence, for antimicrobial activity.^7^ Metal NPs are often coated with charged polymers to increase electrostatic repulsion between the particles and prevent their agglomeration, which would lead to a decrease in their advantageous surface-to-volume ratio and affect their stability.^8−10^ Common coating molecules are chitosan, a polysaccharide positively charged at neutral pH,^11^ and bovin serum albumin (BSA), which is also negatively charged at neutral pH.^12^ The antimicrobial effect of metal NPs is caused by the formation of reactive oxygen species (ROS) and the release of metal ions from the surface of the NPs.^5,13^ Both metal ions and ROS can either diffuse through the membrane, if the NP is interacting extracellularly, or may be released intracellularly after the cellular uptake of the NP.^14^ ROS, such as superoxide (O_2_^–•^), hydrogen peroxide (H_2_O_2_), and hydroxyl radicals (^•^OH), are known to interact with proteins, DNA, or lipids by influencing the activity of enzymes and causing damage in cell membranes or DNA structures.^15−17^

The aggravating problem of increasing antibiotic resistance at decreasing numbers of novel antibiotics fuels the world-wide search for alternative antimicrobial agents.^18^ One of these alternatives could be provided by metal NPs. Metal NPs possess advantageous antimicrobial properties compared to common antibiotics since they usually do not target a single specific cellular mechanism, but rather numerous mechanisms at once in a more unspecific manner. Selenium NPs (SeNPs) are gaining attention in recent years for their potential use as therapeutic agents and many other industrial applications.^19^ Their antimicrobial activity has been reported.^20,21^ However, the cellular and molecular mechanisms which cause the antimicrobial activity and eventually elicit bacterial defense mechanisms have not been investigated. The synthesis of SeNPs or metal NPs in general is performed by chemical reduction of the ionic metal by a reducing agent (such as ascorbic acid or glutathione) and the addition of coating agents to achieve surface functionalization and to prevent agglomeration.^11,12^ Besides the chemical synthesis of SeNPs, the toxic Se forms [selenate (Se^VI^) and selenite (Se^IV^)] can be reduced to less toxic elemental Se (Se^0^) by microorganisms, producing SeNPs with different structures. A few microbes are even capable of modifying Se allotropy of the SeNPs through a crystallization process. For instance, a time-dependent transformation from amorphous (*a*-Se) to crystalline SeNPs [monoclinical (*m*-Se) and trigonal (*t*-Se)] conducted by bacterial species such as *Stenotrophomonas bentonitica* and *Bacillus subtilis* has been proposed.^22−24^ Transformations in the Se structure must be carefully considered as they may involve changes in their physicochemical properties and, therefore, in their potential applications. For example, *t*-Se has indeed been described to be denser rather than amorphous,^25^ which may be beneficial for bioremediation and decontamination purposes due to its higher sedimentation properties.

In this study, we have compared the antibacterial activity of different SeNP types (BSA-coated, chitosan-coated, and undefined coating) on the Gram-negative bacterium *S. bentonitica* and the Gram-positive *Lysinibacillus sphaericus* to silver NPs (AgNPs). The microbial–NP interactions were studied using a combination of complementary techniques. Isothermal microcalorimetry was used to monitor the metabolic response of the bacterial strains to the investigated NPs. Flow cytometry provided a thorough analysis and was applied to determine cytotoxicity, influence on metabolic activity, membrane potential and constitution, and intracellular ROS generation caused by the NPs. Finally, electron microscopy was used to characterize the modes of interaction and NP characterization. Our results demonstrate that commercially available SeNPs outperform AgNPs in terms of their antibacterial activity against the two selected species and might therefore be promising compounds to fight bacterial infections and contaminations in healthcare. The NP envelope appears to have an impact on the interaction as negatively charged SeNPs (primarily UD-SeNPs) seem to enhance the antimicrobial properties. Furthermore, both environmental bacterial species are capable of transforming the initially spherical and amorphous SeNPs into crystalline morphologies, indicating the potential of the bacterial species in bioremediation and crystallization of metal NPs.

## 2 Materials and Methods

### 2.1 Bacterial Strains and Culture Conditions

The bacterial strains *L. sphaericus* NCTC 9602 and *Stenotrophomonas bentonitica* BII-R7 were cultivated in liquid Lysogeny-Broth-medium (10 g/L tryptone, 5 g/L yeast extract, 10 g/L sodium chloride, final pH of 7.0 ± 0.2)^26^ for 24 h at 28–30 °C under constant shaking at 160 rpm. These bacterial model strains were selected to assess the differential influence of NPs on antimicrobial activity considering their distinct cell wall architectures. Both bacterial strains are known for their resistance to environmental stress, such as heavy metal or antibiotic exposure,^27−29^ which make them suitable models for studying bacterial drug resistance. Notably, *S. bentonitica* belongs to the same genus as *Stenotrophomonas maltophilia*, an opportunistic pathogen causing severe infections in humans and is also known for its multi-drug resistance.^30^ Additionally, previous studies have reported a high phosphate (PO_4_^–^), carboxylate (COO^–^), and amine (NH_3_^+^) concentrations in the bacterial cell wall of *S. bentonitica*, revealing its great potential for metallic NP binding,^31^ which is a crucial step for its antimicrobial capabilities.

### 2.2 Metal Nanoparticle Characterization

Selenium and silver nanoparticles (SeNPs and AgNPs) were purchased from Nanocs Inc. (USA). Specifically, three differentially coated SeNPs were purchased, chitosan (CS), bovine serum albumin (BSA), and an undefined coating (UD) at a stock concentration of 0.1 mg/mL (∼1.3 mM). AgNPs had a stock concentration of 0.5 mM.

Dynamic light scattering (DLS) revealed the size distribution and zeta potential of the NPs. The metal NPs were diluted 1:100 in phosphate-buffered saline (PBS; 137 mM sodium chloride, 2.7 mM potassium chloride, and 10 mM phosphate buffer, pH adjusted to 7.2 with 1 M hydrochloric acid) and filled into suitable disposable folded capillary zeta-potential cells (Malvern Panalytical, United Kingdom). The DLS measurements were performed with a Zetasizer NanoZS (Malvern Panalytical, United Kingdom) at 25 °C measuring in the backscatter mode (173°) and with automatic measurement duration (at least 10 consecutive measurements). Before performing the experiments, stock NP solutions were sonicated in ice-cold water for around 10 min. All the samples were prepared in triplicate.

The NPs were further analyzed by electron microscopy to verify size, morphology, and stability of the NPs. To prepare the metal NPs for TEM measurements, 30 μL of each sample was added on a copper grid with a carbon support film. Excess liquid was allowed to evaporate at room temperature (RT) for roughly 20 min. All the samples were analyzed with a transmission electron microscope LIBRA PLUS 120 (Carl Zeiss AG, Germany) equipped with an Oxford Inca 350 energy-dispersive X-ray (EDX) detector (Oxford Instruments, United Kingdom) and a voltage of 200 kV. To obtain size distribution data, more than 200 NPs from at least three different areas were studied regarding their diameter and evaluated with the help of Fiji software by ImageJ.^32^ Moreover, a sample of UD-SeNPs was separately prepared to study the elemental composition of their undefined coating. In particular, high-angle annular dark-field scanning transmission electron microscopy (HAADF-STEM) imaging and spectrum imaging analysis based on EDX were performed at an accelerating voltage of 200 kV using a Talos F200X microscope equipped with a Super-X EDX detector system (FEI, USA).

Moreover, inductively coupled plasma mass spectrometry (ICP–MS) was performed to study putative free metal ions in the supernatant of the NPs. In each case, 300 μL of the NP solution was filled into Vivaspin6 centrifugation tubes (5000 Da molecular-weight cut-off) and centrifuged for 20 min at 4 °C and 5000 rpm. The flow-through was diluted 1:10 in distilled water and subsequently analyzed via ICP–MS with a NexION 300D (PerkinElmer, USA). As a control, untreated NP solutions were also analyzed. All the experiments were performed in triplicate.

### 2.3 Antibacterial Activity Assays

For antibacterial activity experiments, the bacterial pre-cultures were washed three times with PBS via centrifugation at 3000 rpm for 10 min at 4 °C and finally diluted in PBS to an optical density at 600 nm (OD600) of 0.3 (∼0.1 × 10^9^ cells/mL for *L. sphaericus* and ∼1.8 × 10^9^ cells/mL for *S. bentonitica*). The OD600 was measured with the spectrophotometer GENESYS 10S UV–vis (Thermo Fisher Scientific Inc., USA). 1 mL of the samples was inoculated with AgNPs (0.5 mM stock) and SeNPs (1.3 mM stock) to final concentrations between 0 (control) and 100 μM in triplicates. The samples were incubated at 28 °C and 160 rpm for 3, 24, or 48 h.

### 2.4 Microcalorimetry

For isothermal microcalorimetry measurements, washed bacterial cultures of *L. sphaericus* and *S. bentonitica* were exposed to 100 μM SeNPs (BSA-SeNPs, UD-SeNPs, and CS-SeNPs) and 100 μM AgNPs in PBS to a final volume of 2 mL in 4 mL glass ampoules. Untreated cell cultures were used as controls. The metabolic heat flow was simultaneously monitored by a TAM III (Waters GmbH, Germany) over 48 h at 25 °C. All the measurements were performed in triplicate. Maximal metabolic activities were calculated and normalized by the value of the control (0 μM metal NPs), as described by Sachs et al. (2017).^33^

### 2.5 Flow Cytometry

Bacterial samples of *L. sphaericus* and *S. bentonitica* containing metal NP concentrations between 0 and 100 μM were divided into four equal fractions and diluted 1:2 in PBS to study cell viability, membrane potential, intracellular ROS, and DNA content by fluorescence-based flow cytometry (FFC). In all the cases, both untreated and dead cell cultures (by heating at 80 °C in a water bath for 2 h) were used as controls. The cells were collected by centrifugation (11,000*g*, 4 °C, 5 min) after 3, 24, and 48 h and prepared following the instructions of Ruiz-Fresneda et al. (2019).^34^ All the used dyes were stored in stock solutions at −20 °C in the dark, and the stained samples were stored for a maximum of 2 h prior FFC measurement at 4 °C in the dark.

For cell viability and metabolic activity, propidium iodide (PI) and fluorescein diacetate (FDA) were used as suitable fluorescent dyes for FFC.^35^ PI is considered as a marker for non-viable cells since it enters cells with disrupted membranes or increased permeability, subsequently binding intracellular DNA.^36^ The hydrophobic molecule FDA is a measure for metabolic activity since it passively diffuses through the membrane and is a substrate for intracellular esterases, forming the fluorescent fluorescein product.^37^ PI and FDA were added to a final concentration of 3 μM (1.5 mM stock solution in PBS) and 5 μM [1.5 mM stock solution in dimethyl sulfoxide (DMSO)], respectively.

Bacterial membrane potentials were measured by the fluorescent dye 3,3′-dihexyloxacarbocyanine iodide [DiOC_6_(3)], which binds to polarized membranes of active cells.^38^ The dye was added to each sample fraction to a final concentration of 0.2 μM (1 mM stock solution in DMSO). The fluorescent dye 2′,7′-dichlorodihydro-FDA (DC-FDA) was used to monitor ROS formation.^39,40^ Like FDA, DC-FDA is membrane-permeable and becomes deacetylated by intracellular esterases to a membrane-impermeable product. The latter is oxidized by ROS, especially hydrogen peroxide, forming the fluorescent product.^41^ DC-FDA was added to a final concentration of 20 μM in each sample fraction (10 mM stock solution in DMSO).

The intracellular DNA content was accessed by acridine orange (AO), a DNA- and RNA-intercalating fluorescent dye following the protocol of Darzynkiewicz (1990),^42^ with minor changes. Briefly, the sample fraction was initially centrifuged for 20 min at 3000 rpm. The supernatant was discarded, and 250 μL each of ice-cold buffer I (0.1 mM ethylenediamine tetraacetic acid-EDTA, 20 mM citrate phosphate, 0.2 mM sucrose, and 0.1 mL/100 mL Triton-X100; pH 3) and buffer II (10 mM citrate phosphate, 0.1 mM sodium chloride, 0.02 mg/mL AO; pH 3.8) was added, and the sample was gently vortexed. AO was freshly added to the solution of buffer II from a 2 mg/mL stock solution (in distilled water, stored at −20 °C).

All the measurements were performed with a Becton Dickinson FACSCanto II (USA), equipped with forward scatter and side scatter detectors, a 488 nm solid-state, diode laser (air-cooled, 20 mW) and three fluorescence detector filters (530/30 and 616/23 nm), used depending on the incorporated fluorescent dye: 530/30 nm for FDA, DC-FDA, AO-DNA, and DiOC6 (3); 616/23 nm for PI.

### 2.6 Environmental Scanning Electron Microscopy and STEM of Particle–Cell Interactions

The morphology, structure, and location of the NPs after interaction with the bacterial models were analyzed by environmental scanning electron microscopy (ESEM) and STEM. The cells of *L. sphaericus* and *S. bentonitica* were incubated with BSA-SeNPs, UD-SeNPs, CS-SeNPs, and AgNPs separately, to a final concentration of 100 μM for 24 h at 160 rpm and 28 °C. Once again, the untreated cell cultures served as controls.

For ESEM analysis, sample preparation was performed, as described by Ruiz-Fresneda et al. (2018)^23^ with minor modifications. Analysis was performed with a Quanta 400 instrument (FEI, USA) equipped with an EDX detector and a backscattered electron detector. Secondary electrons were excited at 5 kV and a working distance of 6–8 mm.

For STEM analysis, ultra-thin sections of the samples were prepared following the procedures of Merroun et al. (2005).^43^ The analysis of the samples was performed with a transmission electron microscope Titan G2 (FEI, USA), operated at 300 kV and equipped with a Super-X EDX detector, and a high-angle annular dark-field detector (HAADF). Structural characterization of the Se nanostructures was performed by high-resolution TEM combined with selected-area electron diffraction (SAED).

## 3 Results and Discussion

### 3.1 Characterization of Synthetic Selenium and Silver Nanoparticles

Differentially coated SeNPs (BSA, UD, and CS) were characterized by TEM, DLS, and ICP–MS to study the influence of the diameter, zeta potential, shape, morphology, and released metallic ions on their antibacterial activity. Uncoated AgNPs were selected as a reference because they are among the most intensely studied antimicrobial NPs.

TEM analysis indicated similar diameters of between 73 and 86 nm for all the SeNPs, while the uncoated AgNPs were smaller in diameter with 58 nm on an average (Table 1). The results obtained agree with the expected diameter of both Se and AgNPs according to the manufacturer Nanocs Inc. (∼50 nm). The difference in diameters between Se and AgNPs is attributable to the coating of the SeNPs, as exemplarily visible for UD-SeNPs in the Supporting Information (Figure S1). Regarding the hydrodynamic diameters measured by DLS, the SeNPs show comparable values between 126.9 and 137.7 nm (Table 1). TEM images, SAED, and EDX spectra confirmed that the NPs were spherical in shape and consisted of Se with an amorphous nature (Figures S1 and S2) (SAED patterns not shown: due to technical problems, it was not possible to record them). BSA- and CS-coated SeNPs are well-defined, whereas the supplier of the UD-SeNPs did not report the composition of their coating. EDX-elemental mapping analysis of UD-SeNPs showed nitrogen and sulfur signals (Figure S1). This could indicate a coating with a peptide like glutathione, which is a widely used coating and stabilizing agent for metal NPs.^12,44^ Regarding the AgNPs, TEM analysis confirmed their spherical shape and Ag content, as indicated by EDX-elemental characterization (Figure S2).

**Table 1:** Diameter, Hydrodynamic Diameter, and Zeta-Potential Characterization of Differentially Coated SeNPs and AgNPs^a^

			zeta potential (mV)	
nanoparticle	diameter (nm)	hydrodynamic diameter (nm)	distilled water	PBS
BSA-SeNPs	85 ± 27	126.9 ± 3.9	–34.5 ± 0.8	–14.7 ± 1.1
UD-SeNPs	86 ± 21	129.0 ± 5.5	–26.6 ± 1.7	–5.8 ± 0.7
CS-SeNPs	73 ± 29	137.7 ± 6.7	+24.4 ± 3.5	+1.3 ± 0.4
AgNPs	58 ± 16	81.8 ± 1.1	–29.8 ± 2.9	–6.4 ± 0.6

a Diameter was measured by TEM, while the hydrodynamic diameter and zeta potential were measured by DLS.

The zeta potential of all the NPs was measured by DLS in PBS, the selected medium for the antibacterial activity assays, and distilled water, the medium for long-term storage (Table 1). The surface charge measured in distilled water was negative for BSA-SeNPs (−34.5 mV), UD-SeNPs (−26.6 mV), and AgNPs (−29.8 mV) and positive for CS-SeNPs (+24.4 mV). Generally, the ions in PBS seemed to neutralize the zeta potential attributable to an increased salt concentration in PBS.^45,46^ However, even using PBS instead of water to dissolve the NPs, BSA-SeNPs, UD-SeNPs, and AgNPs remained negatively charged, while CS-SeNPs stayed positively charged (Table 1). Cao et al. (2019)^47^ reported that the positive surface charge provided by CS could be mainly attributed to NH_3_^+^ groups, while the net negative charge in BSA-coated might be due to a higher proportion of COO^–^ groups. The obtained zeta potential and diameter values were similar to those reported in previous studies for SeNPs with demonstrated potential for medical applications.^47−49^ Our characterization experiments suggest that both the stability and size of the SeNPs used here seem to be optimal for biomedical applications.

Another parameter that has been previously discussed to affect antimicrobial properties is the release of metal ions from NPs in solution, which may themselves have toxic effects. To investigate potential free metal ions, spontaneously released from the NPs, the supernatant of the stock solution was measured. A maximum of about 10% of the total solution consists of free metal ions for AgNPs and BSA-coated NPs, but lower amounts were found for CS- and UD-coated, with 5.8 and 3.6% of metallic ions released, respectively (Figure S3).

### 3.2 Antibacterial Effect of Selenium and Silver Nanoparticles

#### 3.2.1 Decreased Metabolic Activity in Both Bacteria

**Figure 1:**
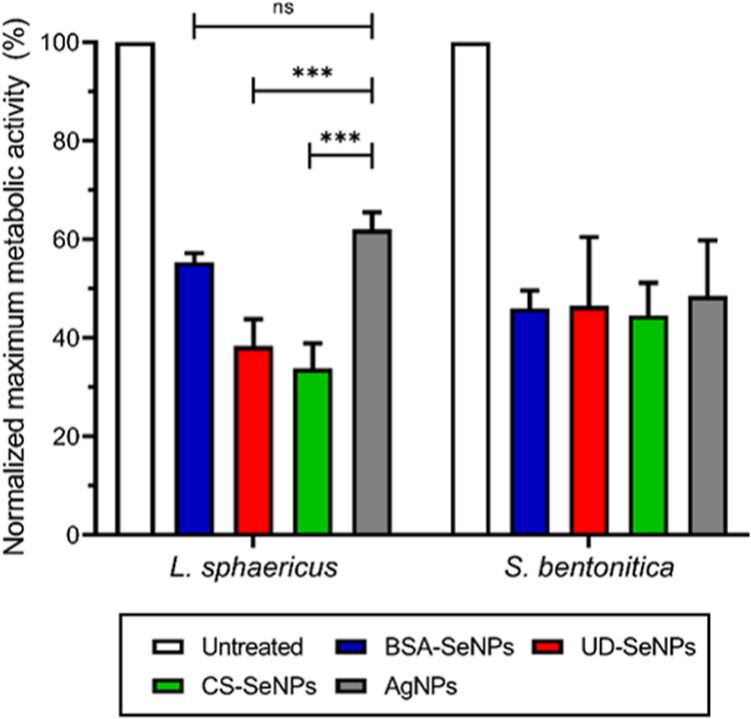
Maximum metabolic activity of *L. sphaericus* and *S. bentonitica* treated with 100 μM metal NP, normalized by an untreated control. The maximum metabolic activity was determined between 2 and 8 h after treatment and corresponds to the maximum heat flow, measured by microcalorimetry. Ordinary one-way ANOVA statistical test was performed with α = 0.05. ns represents non-significant (*p* > 0.05); *** represents *p* < 0.0005.

Microcalorimetric analysis of heat flow has been shown to reveal metabolic activity and growth rate of prokaryotes and eukaryotes in the presence of heavy metals.^33,50,51^ Since metallic NPs are known for their toxic properties, we have exploited microcalorimetry to elucidate putative differences between different coatings of SeNPs and included AgNPs as a control. Maximal metabolic activity of both Gram-positive *L. sphaericus* and Gram-negative *S. bentonitica* was observed between 2 and 8 h after treatment with 100 μM NPs and normalized by the measured maximum metabolic activity, i.e., the maximum heat flow, of the untreated control (Figure 1 and Table 2).

**Table 2:**
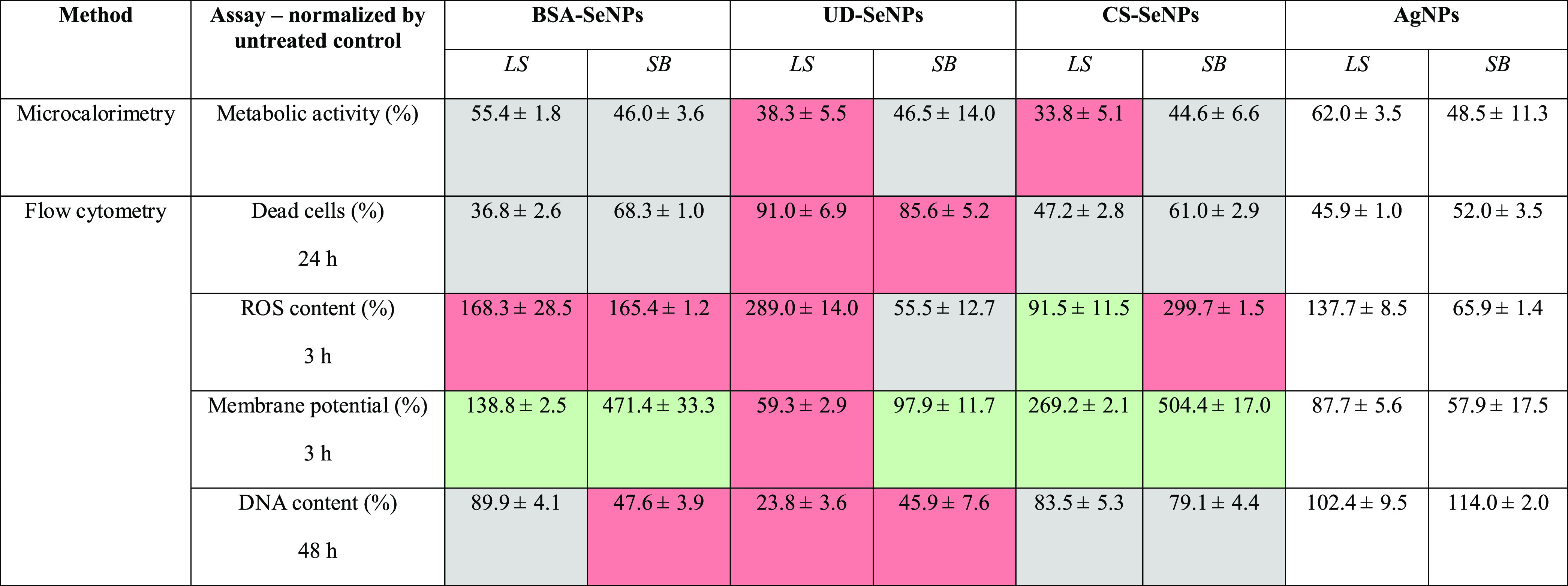
Summary Table Showing the Effect of Se- and AgNPs (100 μM Concentration) on the Metabolic Activity, Percentage of Dead Cells, ROS and DNA Content, and Membrane Potential of *L. sphaericus* and *S. bentonitica* Cells^a^

a LS = *L. sphaericus*. SB = *S. bentonitica*. In red ■ toxicity/stress increased by 20% (compared to AgNPs). In green ■ toxicity/stress decreased by 20% (compared to AgNPs). In gray ■ non-different (compared to AgNPs).

All the investigated NPs reduced the maximal metabolic activity to at least 61% (*L. sphaericus*) and 50% (*S. bentonitica*). For *L. sphaericus*, AgNPs decreased the metabolic activity to 62%, followed by BSA-SeNPs with 55%. UD-SeNPs led to a reduction of 38%, which was only outcompeted by CS-SeNPs with 33% metabolic activity compared to the untreated control. In the case of *S. bentonitica*, AgNPs and SeNPs decreased the metabolic activity below 50% with minor differences, and again the CS-SeNP treatment showed the lowest metabolic activity of 45%. These results suggest that the influence of coating of the SeNPs on the metabolic activity is negligible in the Gram-negative *S. bentonitica*. However, for the Gram-positive *L. sphaericus*, a significant influence of elemental composition and coating on metabolic activity was observed with CS-SeNPs and UD-SeNPs, reducing metabolic activity the most. Significantly, the maximum heat flow only reflects the metabolizing cells but does not detect the possible viability of cells with arrested metabolism. Therefore, flow-cytometric studies were performed to elucidate the toxic effect of SeNPs on different biological parameters, including cell viability.

#### 3.2.2 Selenium Nanoparticles Cause Increased Toxicity to Both Bacteria by Multiple Mechanisms

**Figure 2:**
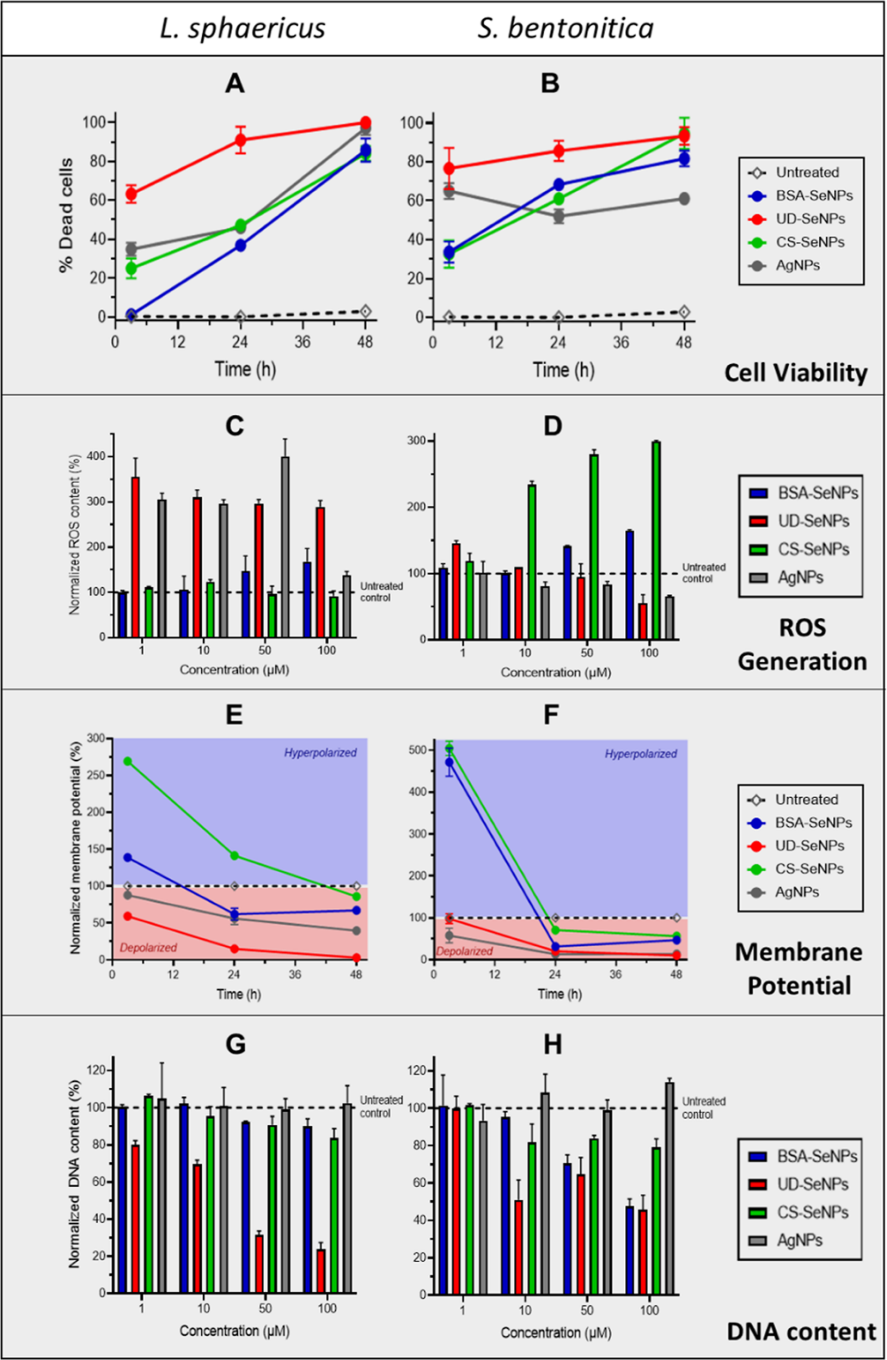
Ratio of dead cells over time of *L. sphaericus* (A) and *S. bentonitica* (B) in contact with the tested NPs relative to the respective untreated control (NP concentration: 100 μM). ROS content generated by *L. sphaericus* (C) and *S. bentonitica* (D) after 3 h’s exposure with NPs at different concentrations (1, 10, 50, and 100 μM). Membrane potential as a measure of membrane polarization over time of *L. sphaericus* (E) and *S. bentonitica* (F) after exposure to the tested NPs at 100 μM concentration. The intracellular DNA content of *L. sphaericus* (G) and *S. bentonitica* (H) cells after 48 h in contact with the tested NPs at different concentrations (1, 10, 50, and 100 μM). Tested NPs: AgNPs (gray), BSA-coated SeNPs (blue), UD-SeNPs (undefined coating in red), and chitosan-coated (CS-SeNPs in green).

Metal NPs were reported to generate ROS, which subsequently interfere with vital cellular mechanisms such as enzymatic activity or may damage cellular constituents such as DNA, ultimately leading to apoptosis.^5,13^ Therefore, cell viability, ROS production, and DNA content were used as parameters for measuring antibacterial activity of the selected SeNPs through flow-cytometry. In all the cases, the conditions used for the experiments included three different contact times between the SeNPs and the two bacterial models (3, 24, and 48 h) and different NP concentrations, ranging from 1 to 100 μM (Table 2, Figures 2, S4–S7).

The ratio of dead cells was measured as the PI uptake and simultaneous reduction in the FDA signal, compared to the viable cells (untreated control). Overall, the metallic NPs showed a concentration-, time-, and composition-dependent toxicity behavior (Figures 2A,B and S4). Among all the SeNPs, UD-SeNPs appeared to be the most toxic during the first 24 h at 100 μM concentration by killing more than 80% of both bacterial species (Figure 2A,B). After 48 h, the percentage of dead cells increased to 100% for *L. sphaericus* and over 90% for *S. bentonitica*. For *L. sphaericus*, the other types of NPs assayed reached similar percentages around 80–90% of dead cells after 48 h incubation (Figure 2A), indicating no significant differences between Se and AgNPs. For *S. bentonitica*, BSA- and CS-SeNPs obtained values close to those obtained for UD-SeNP (80–90% dead cells) after 48 h as well. However, over the same time, AgNPs caused 60% lethality (Figure 2B). These results clearly indicate a higher bactericidal effect of SeNPs for *S. bentonitica* in comparison to AgNPs. Concentration-dependent NP toxicity is displayed in Figure S4. Increasing concentration caused increasing toxicity throughout all samples, except BSA-SeNPs for *S. bentonitica* (Figure S4). Again, UD-SeNPs led to the highest number of dead cells for both species at different concentrations (Figure S4). Our results showed a higher bactericidal role than others reported in the literature. For instance, chemical SeNPs produced with ascorbic acid and polyvinyl alcohol (PVA) inhibited low percentages of cell viability in *Staphylococcus aureus* (Gram-positive) and showed no toxicity against *E. coli* (Gram-negative) by means of flow cytometry.^52^ Other authors, however, found that SeNP-ε-PL (coated with ε-poly-l-lysine) exhibited high antibacterial efficacy regardless of the cell type (gram – or +) based on the minimum inhibitory concentration (MIC) determination.^53^

The intracellular ROS content was studied after 3 h since the used fluorescent dye DC-FDA is dependent on metabolic activity and cell integrity,^41,54^ which is significantly decreased at later exposure times as proven by microcalorimetry. As can be seen in Figure 2C,D, in general terms both the SeNPs and AgNPs generated an excess of ROS in the tested bacterial cells, highlighting the role of the NPs as important stress agents. In more detail, BSA- and CS-coated SeNPs seemed to generate lower amounts of ROS in *L. sphaericus* (Figure 2C), which agrees with the slightly lower influence of these NPs on cell viability (Figure 2A). Indeed, the results showed very similar values in comparison to the untreated controls, indicating insignificant ROS production after interaction with these NPs. On the other hand, the production of ROS was significantly increased when the cells of *L. sphaericus* were in contact with UD-SeNPs and AgNPs, which is also in line with the cell viability test in Figure 2A, where these NPs led to the highest proportion of dead cells. As expected according to the cell viability tests for *S. bentonitica* (Figure 2B), the SeNPs generated higher ROS content than the AgNPs (Figure 2D). However, the ROS amounts produced were very low or even lower than the untreated controls when the cells were in contact with UD-NPs, suggesting that the effect exerted by these NPs on cell viability (Figure 2B) was not related to ROS generation. The lower amount of ROS maybe caused by an already reduced cell viability (up to 78% of dead cells after 3 h at 100 μM) (Figure 2B) as well as depolarization, and thus, the disruption of the cell membrane. Only CS-NPs seemed to significantly increase the ROS generation in *S. bentonitica* cells, probably inducing cellular death as observed in Figure 2B. The results suggest that, depending on the NP type, different mechanisms may be involved in the antimicrobial activity. A few studies have already reported that chemically synthesized SeNPs coated with different compounds (PVA, ε-poly-l-lysine, etc.) also promoted ROS production in several bacterial species, such as *S. aureus*, *Enterococcus faecalis*, and *E. coli*, and demonstrated their potential as a broad-spectrum antimicrobial material in medical applications.^7,53^

Polarization of the bacterial membranes, a probe for cell integrity and stress, was studied via flow-cytometry using DiOC_6_ (3). In Figure 2E,F, the membrane potential normalized to the untreated control (100%) is displayed over 48 h at a NP concentration of 100 μM. The highest membrane potential was observed at early time points (first 12 h) for both bacteria. Specifically, BSA-SeNPs and CS-SeNPs led to a hyperpolarization of the cells, indicating a massive stress reaction. At later time points (after 24 h), the membrane polarization decreased to even below the value of the untreated control for all the tested NPs, indicating membrane disruption and cell death (Figure 2E,F). UD-SeNPs and AgNPs cause the depolarization of the cell membranes of *L. sphaericus* and *S. bentonitica* from the beginning, resulting in the disruption of cell membrane function faster than the rest of NPs assayed. Lower NP concentrations generally caused less differences in polarization compared to the untreated control (Figures S5 and S6), confirming the former results of a time- and concentration-dependent toxicity of the metal NPs.

As a fourth parameter, the intracellular DNA content of bacterial cells exposed to metal NPs was measured by AO at different time intervals (3, 24, and 48 h). After 3 h, no significant differences in the DNA content were observed for any NPs assayed (Figure S7A,B) but increasing exposure times to 24 h (Figure S7C,D) and 48 h led to a general decrease in the DNA content (Figure 2G,H). UD-SeNPs caused the highest effect in the DNA content compared to the untreated control and the rest of the NPs with a considerable decrease of up to 24% (*L. sphaericus*) and 46% (*S. bentonitica*) after 48 h and 100 μM (Figure 2G,H). Hence, particularly the most toxic UD-coated SeNPs also seem to affect DNA content or interfere with the DNA structurally. Overall, the other SeNPs (BSA-and CS-coated) seem to play a lesser influence on DNA damage but still led to a decrease of up to 48 and 79% after 48 h at 100 μM, respectively. However, no significant decrease in the DNA content was observed for AgNPs at any time or concentration, indicating a higher toxicity of SeNPs compared to the AgNPs. This technique has been used to measure DNA damage produced by SeNPs in different carcinoma cell lines and assess their anti-tumoral properties.^55,56^ However, as far as we know, this is the first time it has been used with bacteria to assess the antimicrobial properties of SeNPs.

To sum up, cell viability of *S. bentonitica* was most severely and strongly reduced by SeNPs (especially by UD-SeNPs) than that by AgNPs, while the viability of *L. sphaericus* was equally decreased by both the Se (mainly UD-SeNPs) and AgNPs. Accordingly, ROS production was significantly increased when *S. bentonitica* cells were in contact with SeNPs, particularly CS-SeNPs. For *L. sphaericus*, the highest ROS generation was observed in the presence of UD-Se and AgNPs. Se (mainly UD-SeNPs) and AgNPs led to strong membrane depolarization for both bacteria. Finally, a higher DNA degradation for both bacterial species was detected in the presence of SeNPs in comparison to AgNPs. Once again, UD-SeNPs stands out as the most toxic NP type with the highest DNA degradation values. Overall, our results indicate better antimicrobial properties of SeNPs compared to conventionally used AgNPs. Not only are the inhibition levels toward bacteria generally better for SeNPs when compared to metal NPs composed by ZnO, CuO, and TiO_2_, but Se also has some advantages. As an essential trace element, Se is involved in various metabolic and biological functions and shows much lower toxicity against human cells^57^ since it can be metabolized and removed from the body more easily. In addition, synthesis of SeNPs is economical and can be accomplished with the use of biological agents.^58^

Previous findings observed that SeNPs generally have lower antimicrobial efficacy against Gram-negative bacteria. However, our results demonstrate the potential broad spectrum of SeNPs as effective antimicrobial agents for both Gram-negative and Gram-positive bacteria. Among all the SeNPs, the negative charged UD-SeNPs exhibited the highest toxicity levels for both bacterial models in most cases. This may generate some controversy since many papers in the literature state that positively charged NPs could have better antibacterial properties due to their more efficient electrostatic interaction to negatively charged bacterial surfaces.^48,59^ For instance, AgNPs with positive charge exhibited more effective antimicrobial properties than negative and neutral ones against several Gram-negative (*E. coli* and *Proteus vulgaris*) and Gram-positive (*S. aureus*, *Streptococcus mutans*, and *Streptococcus pyogenes*) bacteria.^59^ However, in recent years, several studies have reported negatively charged NPs with powerful antimicrobial activity. The studies of Salvioni et al. (2017)^60^ demonstrated elevated inhibition rates in the growth of *E. coli* MG1665 and *S. aureus* when exposed to AgNPs with a negative charge. Although negatively charged NPs do not exhibit such a strong electrostatic attraction toward bacterial cell walls, our results indicate that UD-SeNPs are still the most toxic. This is probably due to a multi-modal action mechanism of these NPs, which leads to an increased antibacterial activity. Apart from the high rates of ROS production and DNA degradation, our negatively charged UD-SeNPs also caused a major impact on cell membrane disruption. In addition, other molecular and physical mechanisms, such as ATP disruption, thiol depletion, or intracellular penetration, may be involved.^61^ ATP is an important intracellular energy source for living organisms, and its disruption can seriously affect enzymatic reactions of both the respiration and metabolism of bacteria. A significant decrease of intracellular thiol levels in *S. aureus* after exposure to Se nanoclusters has also recently been detected for the first time.^62^ Consequently, these compounds may also have an important role as an antimicrobial action mechanism. Another explanation for the bactericidal capacity of UD-SeNPs could be an increased ability to penetrate the cytosol and the consequent impairment of metabolic functions. Finally, the release of Se ions from the NPs has also been proposed as an antibacterial mechanism. However, our results did not show significant amounts of released ions to represent an important factor for bactericidal activity (Figure S3).

### 3.3 Transformation of the SeNPs by Both Bacteria and the Implication for Potential Applications

ESEM and HAADF-STEM were performed to characterize the interaction of the Ag- and SeNPs with the bacterial model strains *L. sphaericus* and *S. bentonitica* concerning their extra- and intracellular localization. Thereby, 100 μM NPs were incubated with the bacterial cells for 24 h. A control without NPs was investigated as well.

**Figure 3:**
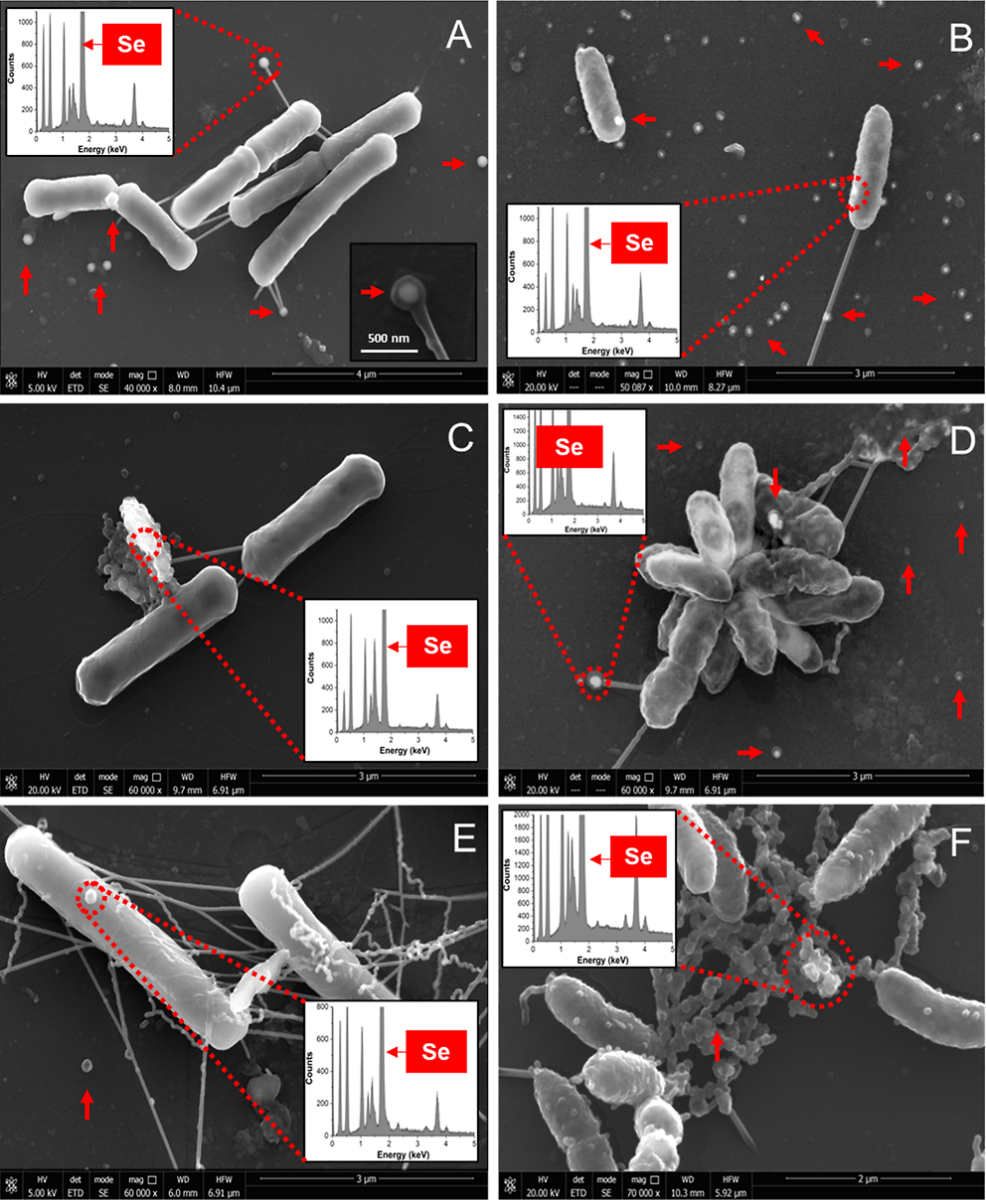
Environmental scanning electron microscopy micrographs of *L. sphaericus* (left panels: A,C,E) and *S. bentonitica* (right panels: B,D,F) incubated for 24 h with 100 μM UD-SeNPs (A,B), BSA-SeNPs (C,D), and CS-SeNPs (E,F), respectively. Elemental composition of the NPs was investigated by EDX (see the insets). Additional SeNPs interacting with the bacterial cells are highlighted by arrows. Scale bars: A (4 μm), B–E (3 μm), and F (2 μm).

ESEM micrographs showed similar interactions of *L. sphaericus* and *S. bentonitica* after exposure to all SeNP types (Figure 3). The overall morphology of the cells and their membranes did not seem to be negatively affected at all with a few exceptions, even compared to the untreated controls (Figure S8). EDX analysis indicated the SeNPs contain Se (insets are shown in Figure 3). Spherical SeNPs were observed mainly interacting with bacterial surfaces, extracellular filaments, and also in the extracellular space in all the cases (Figure 3A–F). These filaments may correspond to flagella-like proteins previously suggested to be involved in SeNP transformation.^23^ The results suggest that the SeNPs exert their toxicity by interacting with the cell surfaces, but not through internalization of the NPs, since no intracellular NPs were found. Once the SeNPs are attached, they most probably cause ROS formation in the cells or lead to other harmful molecular mechanisms, as discussed in the previous section. The extracellular filaments may correspond to flagella-like proteins, previously reported to be involved in the formation of nanomaterials and biofilms.^23,63^ Ruiz Fresneda et al. (2018)^23^ suggested the role of these flagella-like filaments in *S. bentonitica* as a template for SeNP aggregation and transformation to nanorods from nanospheres. Indeed, SeNPs agglomerations were mostly observed in cell cultures with BSA and CS-coated SeNPs after 24 h (Figure 3C–F), suggesting the possible role of these filaments in SeNP aggregation that could lead to NP transformation.

**Figure 4:**
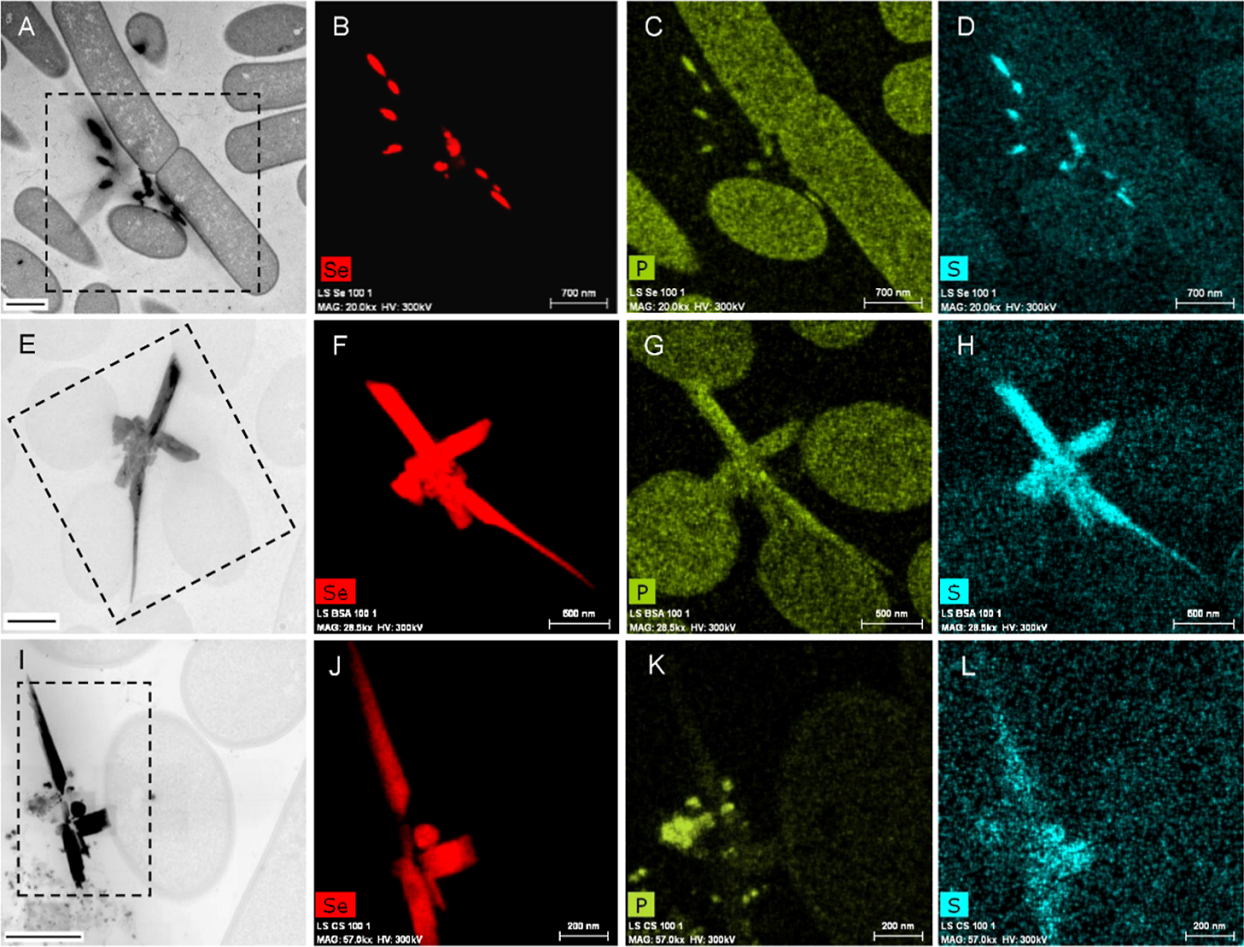
Electron microscopy micrographs of thin sections of *L. sphaericus* treated with 100 μM UD-SeNPs (A–D), BSA-SeNPs (E–H), and CS-SeNPs (I–L) for 24 h, respectively. The dashed boxes highlight the areas which were analyzed by EDX-element mapping, with the results displayed for selenium in red, phosphorus in yellow green, and sulfur in cyan. Scale bars: 700 nm (A–D), 500 nm (E–I), and 200 nm (J–L).

Interestingly, STEM imaging of thin sections combined with EDX-based element mapping of *L. sphaericus* after exposure to UD, BSA, and CS SeNPs showed the presence of different NP shapes including spherical, polygonal, hexagonal-shaped, nanorod-like, and agglomerated formations (Figure 4). Since only Se nanospheres were observed in control measurements of SeNPs without bacteria (Figure S2), our hypothesis is that a transformation from spherical to the different shapes observed (polygonal, hexagonal, and nanowire formations) is conducted by the cells.

**Figure 5:**
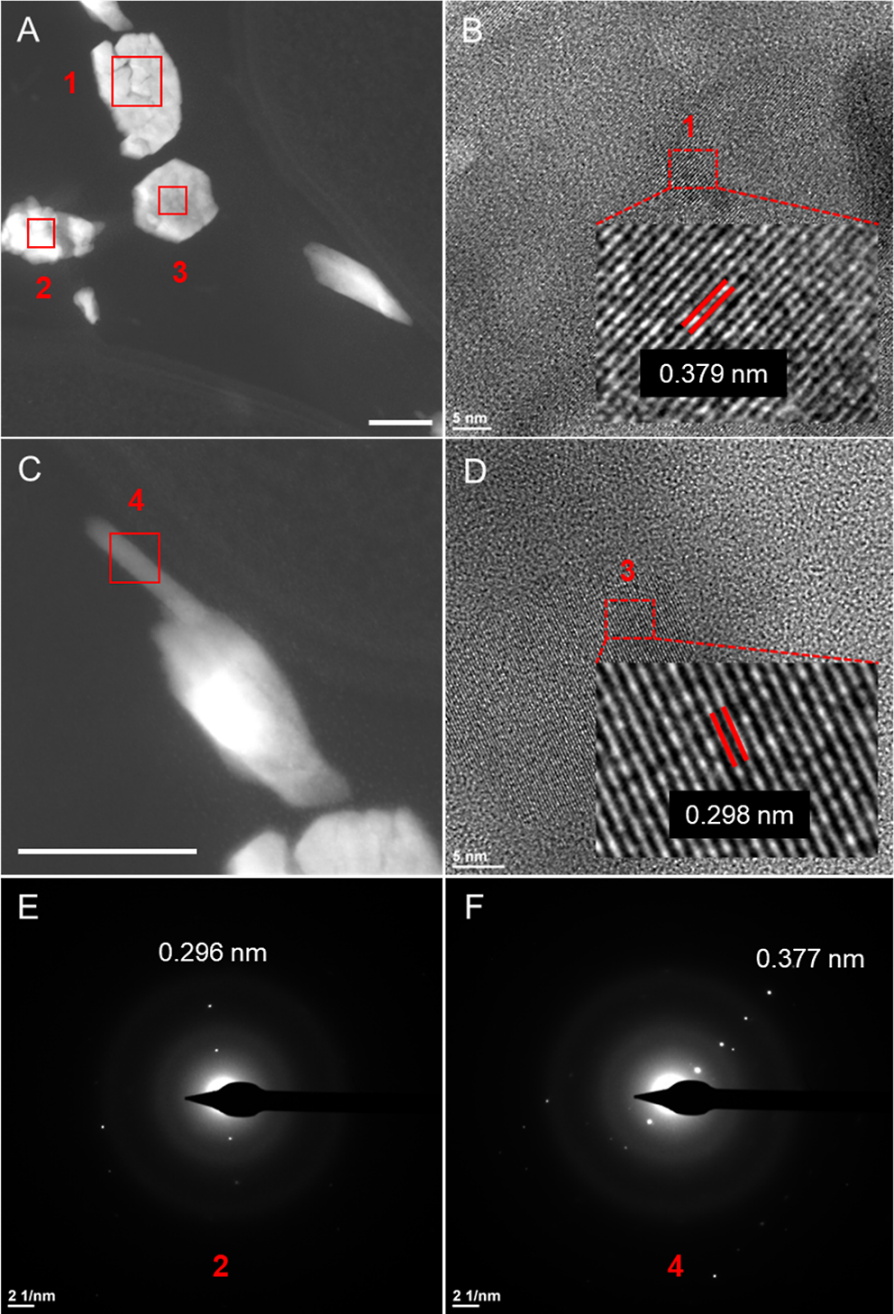
HAADF-STEM images showing selenium nano- and microstructures (A,C) after exposure of *L. sphaericus* to 100 μM UD-SeNPs for 24 h. HR-TEM micrographs (B,D) corresponding to regions 1 and 3, and SAED patterns (E,F) corresponding to regions 2 and 4. Scale bars: A and C (100 nm) and B and D (5 nm).

Diffraction patterns obtained by SAED analysis derived from UD-SeNPs (Figure 5A,C) showed two different lattice spacings of 0.296 and 0.377 nm (Figure 5E,F). High-resolution TEM (HR-TEM) images confirmed the presence of both lattice spacings with very similar values (0.298 and 0.379 nm), which may correspond to different crystal planes of Se (Figure 5B,D) according to the American Mineralogist Crystal Structure Database (http://rruff.geo.arizona.edu/AMS/amcsd.php). Specifically, 0.296 and 0.298 nm are close to the 0.3 nm *d*-spacing, which may correspond to planes (1 0 1) of trigonal Se (*t*-Se) and (2 2 1) of monoclinical (*m*-Se), among other planes. The other *d*-spacings obtained (0.377 and 0.379 nm) match that of 0.37 nm and may also correspond to the plane (1 0 0) of *t*-Se and different planes of *m*-Se. These results suggest the presence of two different crystal structures (monoclinic and trigonal Se) in the transformed NPs and support the hypothesis of a transformation process from amorphous forms to the thermodynamically more stable crystalline allotrope *t*-Se, through the *m*-Se transitional state. Very similar results were obtained for CS and BSA-SeNPs exposed to *L. sphaericus* cells, as shown in Figures S9 and S10, respectively. This indicates a coating-independent transformation mechanism by *L. sphaericus*. Indeed, some SeNPs and mostly agglomerations seem to present a hybrid state between crystalline and non-crystalline, as can be seen in Figure S9A,B. The co-existence of mixed crystal phases in individual NPs has been documented before in Sn NPs.^64^ In the case of AgNPs, no significant differences were observed in the structure, morphology, and size of the AgNPs after contact with both studied strains, *L. sphaericus* and *S. bentonitica* (Figure S11). According to ESEM and STEM micrographs, the cells do not seem to be very damaged, and no changes were apparent in the AgNPs in comparison to the controls (Figure S2).

*S. bentonitica* has been previously demonstrated to crystallize chemical SeNPs from amorphous to monoclinical and trigonal states.^65^ However, here we have demonstrated for the first time that this transformation can also be performed by the cells of *L. sphaericus*. Our findings suggest that the transformation occur extracellularly. First, there is no evidence of intracellular SeNPs, and all of them appeared attached to the cellular membranes or to extracellular filaments. In addition, the SeNPs are prefabricated nanospheres that have been added externally before exposure to the cells. Thus, it is unlikely that SeNPs of this size (73–85 nm) enter the cells before their subsequent release and transformation. When Se (IV) is added as the Se source, however, the cells of *S. bentonitica*, have been previously reported to reduce it to Se^0^ NPs that are produced intracellularly, and after 48 h, they are released to the extracellular space and transformed to crystalline Se nanostructures in a similar way to *L. sphaericus*.^23^ In the above referenced research, no intracellular crystalline SeNPs were found, supporting that transformation only seems to occur in the extracellular space. The possible role of the already mentioned flagella-like protein and extracellular polymeric substances (EPS) in Se crystallization provide a feasible, quick, economical, and environmentally friendly method to produce crystalline SeNPs with a well-known variety of applications.^24^ Different sub-cellular fractions and EPSs of both bacteria have been extracted in our laboratory to evaluate their ability to fabricate crystalline SeNPs.

The ability of *L. sphaericus* and *S. bentonitica* to crystallize SeNPs could play important roles, not only for medical but also for industrial and environmental activities. Crystalline NPs display unique physicochemical properties of extreme interest in certain applications including pharmaceutical, food processing, medical, microelectronics, commercial fields, and other industries. Some studies have demonstrated a higher antimicrobial efficacy of crystalline NPs in comparison to amorphous ones. Specifically, the toxicological potential of silica NPs has been associated to their crystalline properties.^66^ AgNPs have also been reported to have better antibacterial properties in the crystalline state.^67,68^ From an environmental point of view, Se nanocrystals exhibit excellent settling properties which facilitate their immobilization and recovery.^25^ Not only crystallinity but also the size, shape, surface charge, etc. may have implications for the properties SeNPs. Small-sized SeNPs (<50 nm approximately) tend to have lower sedimentation capacity and, therefore, higher mobility.^69^ In addition, it is generally assumed that the antimicrobial properties of NPs are size-dependent, and smaller NPs can interact and enter more easily and thus damage cells.^70^ However, recent results have indicated that BSA-coated SeNPs significantly larger than chitosan-coated SeNPs have a better overall antimicrobial activity.^48^ This emphasizes again that not only size but also a combination of factors influence SeNP properties such as the surface chemistry, charge, cell host type, NPs shape, etc. Regarding the shape of the NPs, some authors have reported that Se nanospheres are less toxic since they are less harmful to cell integrity.^69,71^ In this line, Zhao et al. (2013)^72^ demonstrated that needle-like NPs (similar shape to those we have obtained) of hydroxyapatite cause larger death rates in BEAS-2B cells than spherical-shaped NPs. This may be due to their capacity to damage cells through direct contact.^70^ Consequently, the final rod-shape of our transformed NPs could further promote their antimicrobial properties.

## 4 Conclusions

The results we present demonstrate the antibacterial activity of different SeNP types (UD-, BSA-, and CS-coated SeNPs) against the Gram-positive *L. sphaericus* and the Gram-negative bacteria *S. bentonitica*. Overall, the tested SeNPs showed higher toxicity than the AgNPs. Specifically, negatively charged UD-SeNPs presented the most harmful effects on cell viability, membrane depolarization, and DNA degradation for both types of bacteria, showing their potential bactericide broad spectrum. Everything suggests that antimicrobial activity is likely to be due to a multi-modal mechanism. With the urgent need for novel antibacterial agents, our findings underline the potential of both negatively and positively charged SeNPs as a possible antibacterial alternative for their application in medicine and healthcare in the near future.

An allotropic transition of the prefabricated *a*-Se nanospheres to crystalline *m*-Se and *t*-Se (hexagonal-shaped, polygonal-shaped, and nanorod) was conducted by *L. sphaericus* as a putative detoxification mechanism in a very similar way as previously reported for *S. bentonitica*.^23,73^ The crystallinity, shape, and size of the SeNPs transformed by the cells highlight *L. sphaericus* and *S. bentonitica* as potential bioremediation agents due to the already reported lesser mobility and greater stability of crystalline Se products. This may have a potential influence on environmental decontamination and SeNP crystallization. Thus, we propose *S. bentonitica* and *L. sphaericus* as promising candidates for bioremediation and crystallization of NPs using a novel, biological, and green method.

## Supporting Information Available

HAADF-STEM image and EDX-element distribution showing coating composition of UD-SeNPs; TEM images and their respective EDX spectra of BSA-, UD-, CS-SeNP, and AgNP controls without bacteria; free metal ions released by the Se- and AgNPs measured in supernatant samples by ICP–MS; cell viability rates of *L. sphaericus* and *S. bentonitica* against increasing concentrations of NPs; time- and NP concentration-dependent measurements on the membrane potential of *L. sphaericus* and *S. bentonitica*; DNA content measurements of *L. sphaericus* and *S. bentonitica* over increasing concentrations of NPs at different incubation times (3 and 24 h); SEM images of the bacterial model (*L. sphaericus* and *S. bentonitica*) controls without NPs; HAADF-STEM images of CS-SeNP contacted with *L. sphaericus* cells and high-resolution images showing their crystallization; HAADF-STEM images of BSA-SeNP contacted with *L. sphaericus* cells and high-resolution images showing their crystallization; and SEM images of AgNPs incubated with *L. sphaericus* and *S. bentonitica* and their respective EDX spectra (PDF)

## Abbreviations

NP: nanoparticle
AgNPs: silver nanoparticles
SeNPs: selenium nanoparticles
ROS: reactive oxygen species
(^•^OH): hydroxyl radicals
Se^VI^: selenate
Se^IV^: selenite
Se^0^: elemental selenium
*a*-Se: amorphous selenium
*m*-Se: monoclinical selenium
*t*-Se: trigonal selenium
CS: chitosan
UD: undefined coating
BSA: bovine serum albumin
DLS: dynamic light scattering
PBS: phosphate-buffered saline
EDX: energy-dispersive X-ray
HAADF-STEM: high-angle annular dark-field scanning transmission electron microscopy
ICP-MS: inductively coupled plasma mass spectrometry
PI: propidium iodide
FDA: fluorescein diacetate
AO: acridine orange
ESEM: environmental scanning electron microscopy
SAED: selected-area electron diffraction
