# Supplementary material for: Exploring Antibacterial Activity and Bacterial-Mediated Allotropic Transition of Differentially Coated Selenium Nanoparticles
Source: ACS Appl Mater Interfaces. 2023 Jun 9;15(25):29958–70. doi: 10.1021/acsami.3c05100 (PMC10316328; doi:10.1021/acsami.3c05100)
Supplement: Supplementary file 1 — am3c05100_si_001.pdf [file am3c05100_si_001.pdf]

Supporting Information

–

Exploring antibacterial activity and bacterial-mediated allotropic transition of differentially-coated selenium nanoparticles

Miguel Angel Ruiz-Fresneda[1,#,*], Sebastian Schaefer[1,2,†,#], René Hübner[3], Karim Fahmy[2], Mohamed Larbi Merroun[1]

[1] University of Granada, Department of Microbiology, Campus Fuentenueva, 18071 Granada, Spain

[2] Helmholtz-Zentrum Dresden-Rossendorf, Institute of Resource Ecology, Bautzner Landstraße 400, 01328 Dresden, Germany

[3] Helmholtz-Zentrum Dresden-Rossendorf, Institute of Ion Beam Physics and Materials Research, Bautzner Landstraße 400, 01328 Dresden, Germany

[†] Current address: University of New South Wales, School of Chemical Engineering, New South Wales 2052, Sydney, Australia

[*] Author for correspondence: mafres@ugr.es

[#] **MA. Ruiz-Fresneda and S. Schaefer contributed equally to this manuscript.**

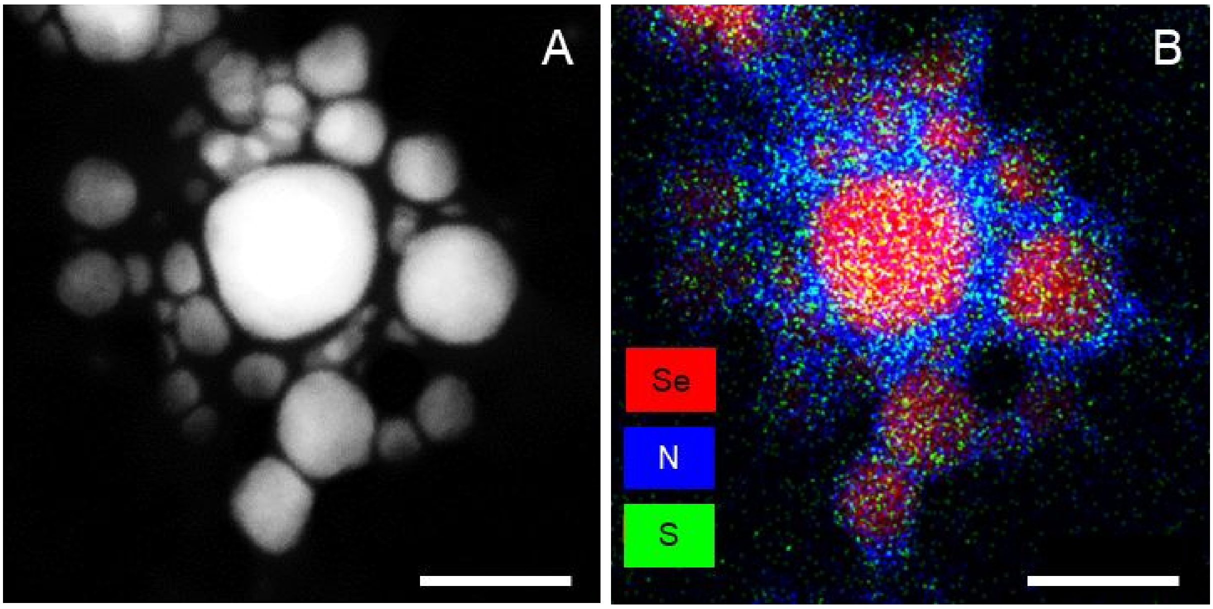

Figure S1. HAADF-STEM image (A) and corresponding EDX-based element distribution mapping (B) of the UD-SeNPs. The Scale bars represent 80 nm.

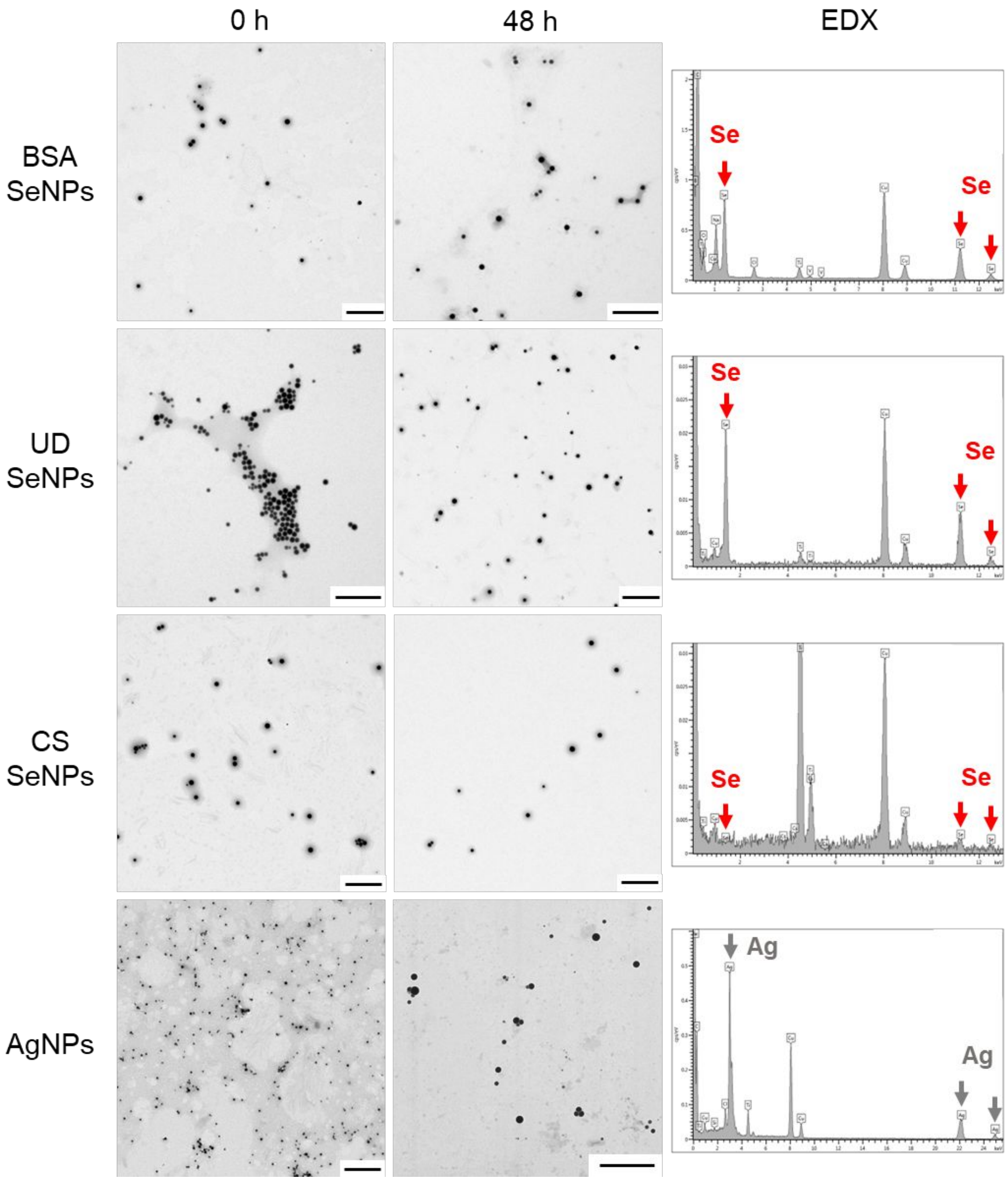

Figure S2. Transmission electron microscopy images and energy-dispersive X-ray (EDX) spectra of BSA-, UD-, CS-SeNPs, and AgNPs in PBS at 0 h and after 48 h incubation at 28 °C. The scales in the microscopy images represent 1 µm. EDX spectra were taken from the 0 h samples to confirm the elemental composition of the metal NPs (Se – red arrows, Ag – grey arrows). The CS-SeNPs were too unstable for EDX measurement, wherefor only a very marginal Se signal was observed.

Note: Besides Se and Ag signals in the EDX spectra, further peaks have been observed that originate from the TEM sample grid (made of copper and with a carbon support film) and the

phosphate buffered saline (PBS) that has been used as a medium for the metal nanoparticles (sodium and chlorine).

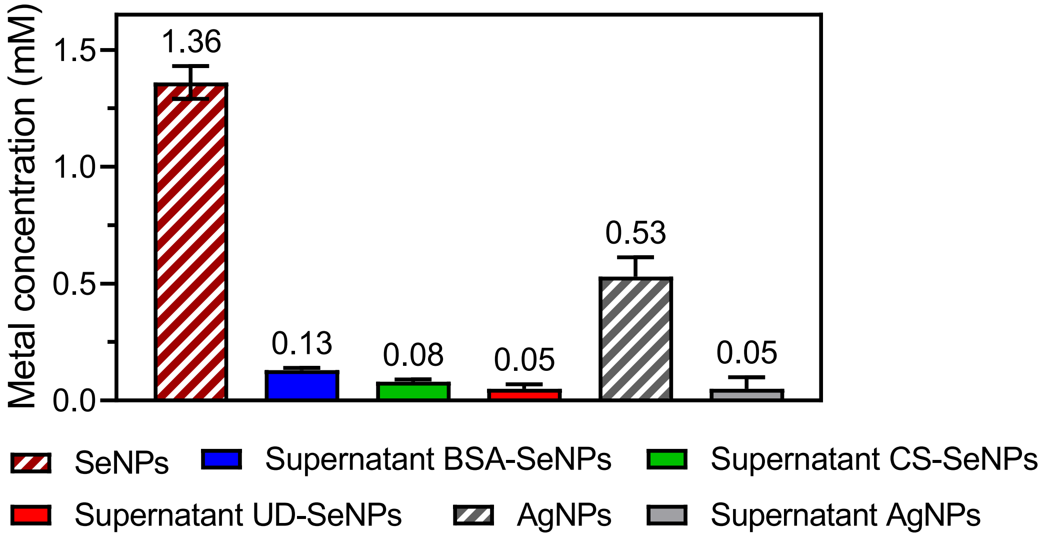

Figure S3. ICP-MS analysis of metal NP solutions (striped columns) and NP supernatant solutions to determine free metal ions in solution.

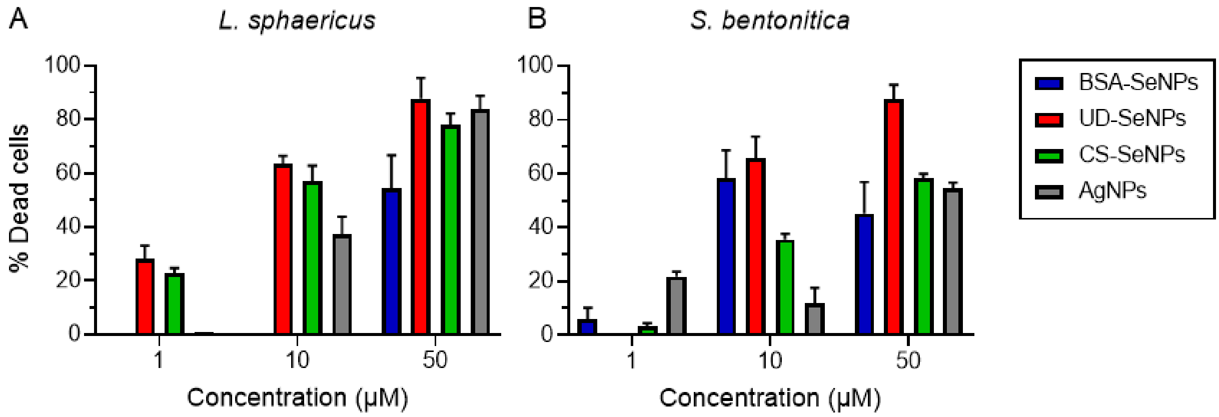

Figure S4. Cell viability rates of *L. sphaericus* (A) and *S. bentonitica* (B) after 48 h at differing NP concentrations (1, 10, and 50 µM) as determined by PI/FDA staining. BSA-coated SeNPs (blue), SeNPs with undefined coating (UD-SeNPs, red), chitosan-coated SeNPs (green), and AgNPs (grey) were investigated and are displayed relative to the respective untreated control.

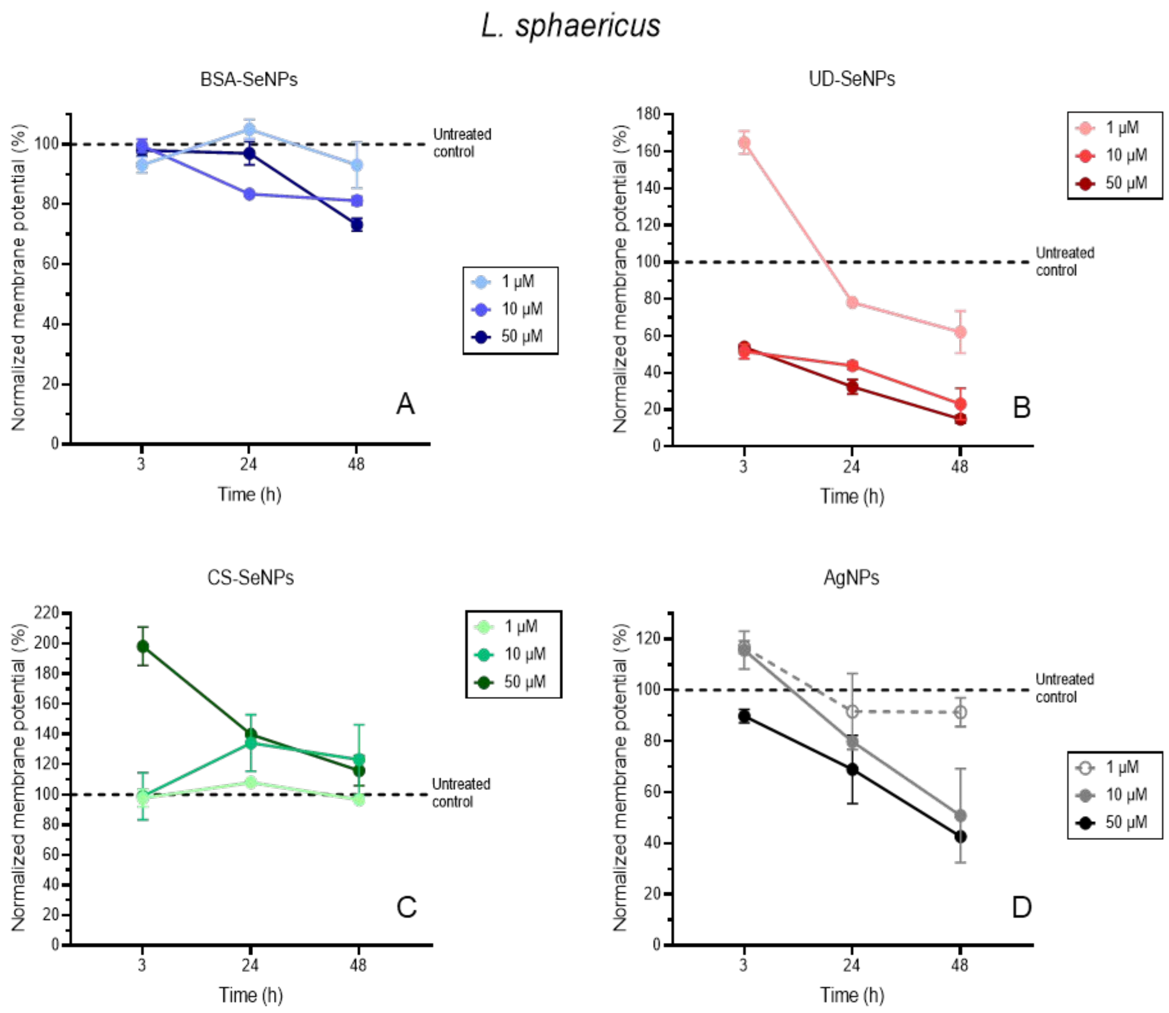

Figure S5. Membrane potential over time of *L. sphaericus* after exposure to BSA-coated SeNPs (A, blue), SeNPs with undefined coating (B, red, UD-SeNPs), chitosan-coated (C, green, CS-SeNPs), and Ag-NPs (D, grey) at concentrations of 1, 10, and 50 μM as determined by $DiOC_6(3)$.

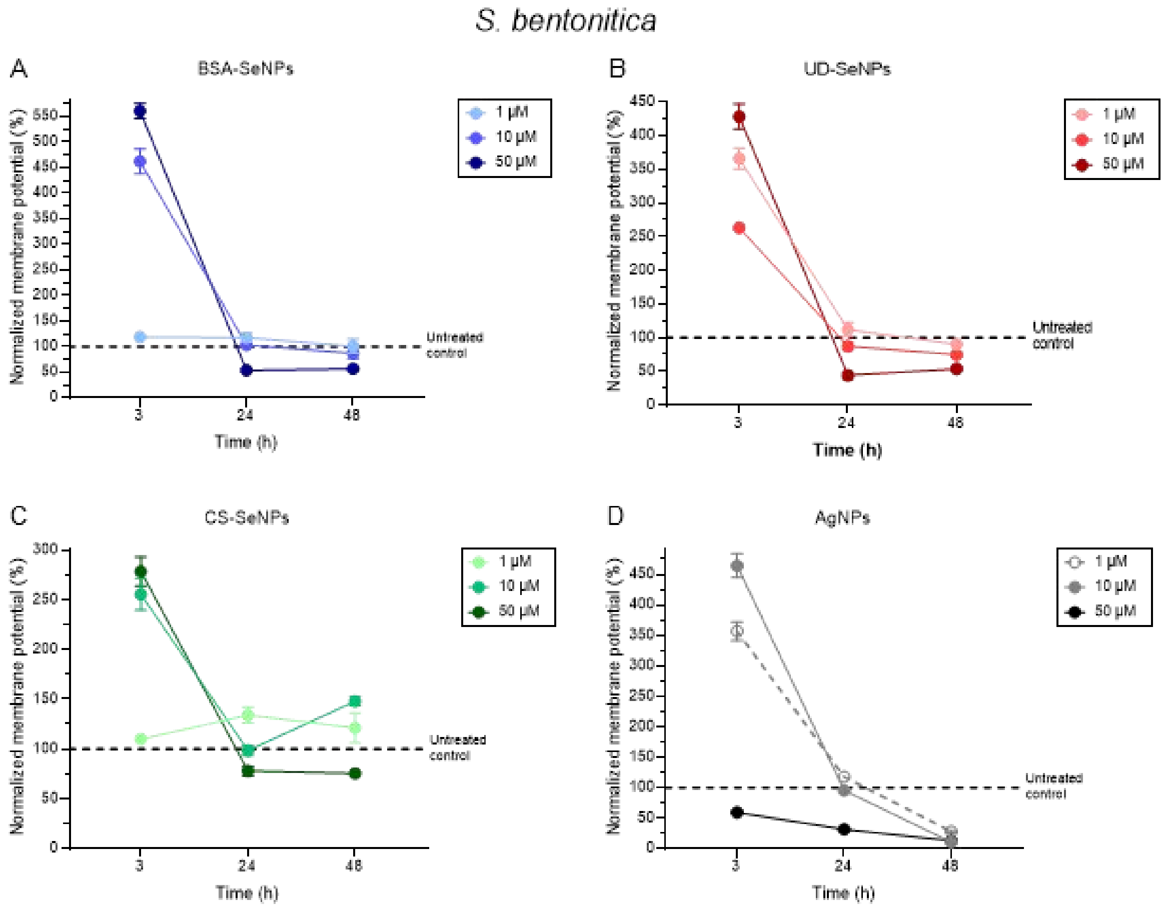

Figure S6. Membrane potential over time of *S. bentonitica* after exposure to BSA-coated SeNPs (A, blue), SeNPs with undefined coating (B, red, UD-SeNPs), chitosan-coated (C, green, CS-SeNPs), and AgNPs (D, grey) at concentrations of 1, 10, and 50 µM as determined by $DiOC_6(3)$.

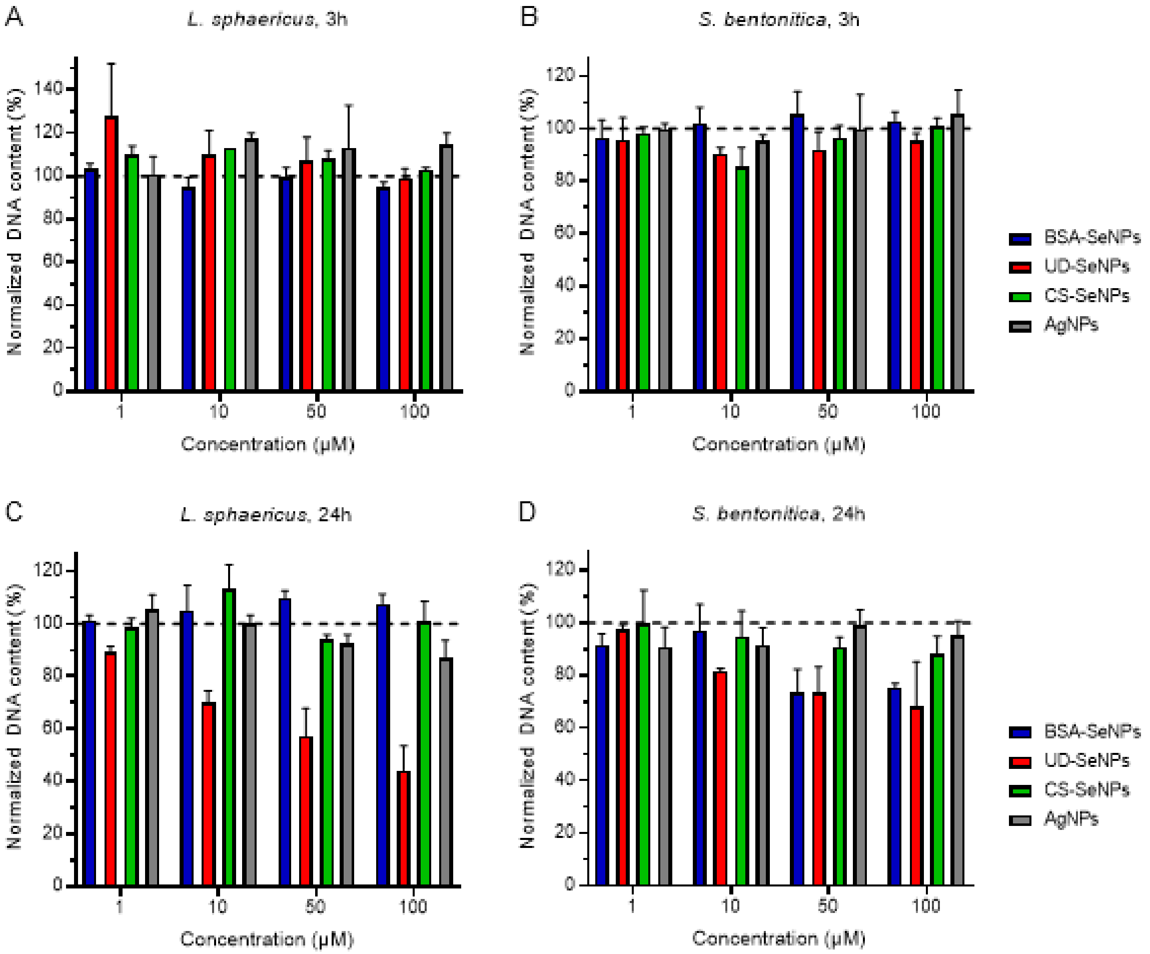

Figure S7. Intracellular DNA content of *L. sphaericus* (A, C) and *S. bentonitica* (B, D) cells after 3 h (A, B) and 24 h (C, D) in contact with AgNPs (grey), BSA-coated SeNPs (blue), UD-SeNPs (undefined coating) (red) and chitosan-coated (CS-SeNPs) (green) at different NP concentrations (1, 10, 50, and 100 μM) determined by acridine orange staining.

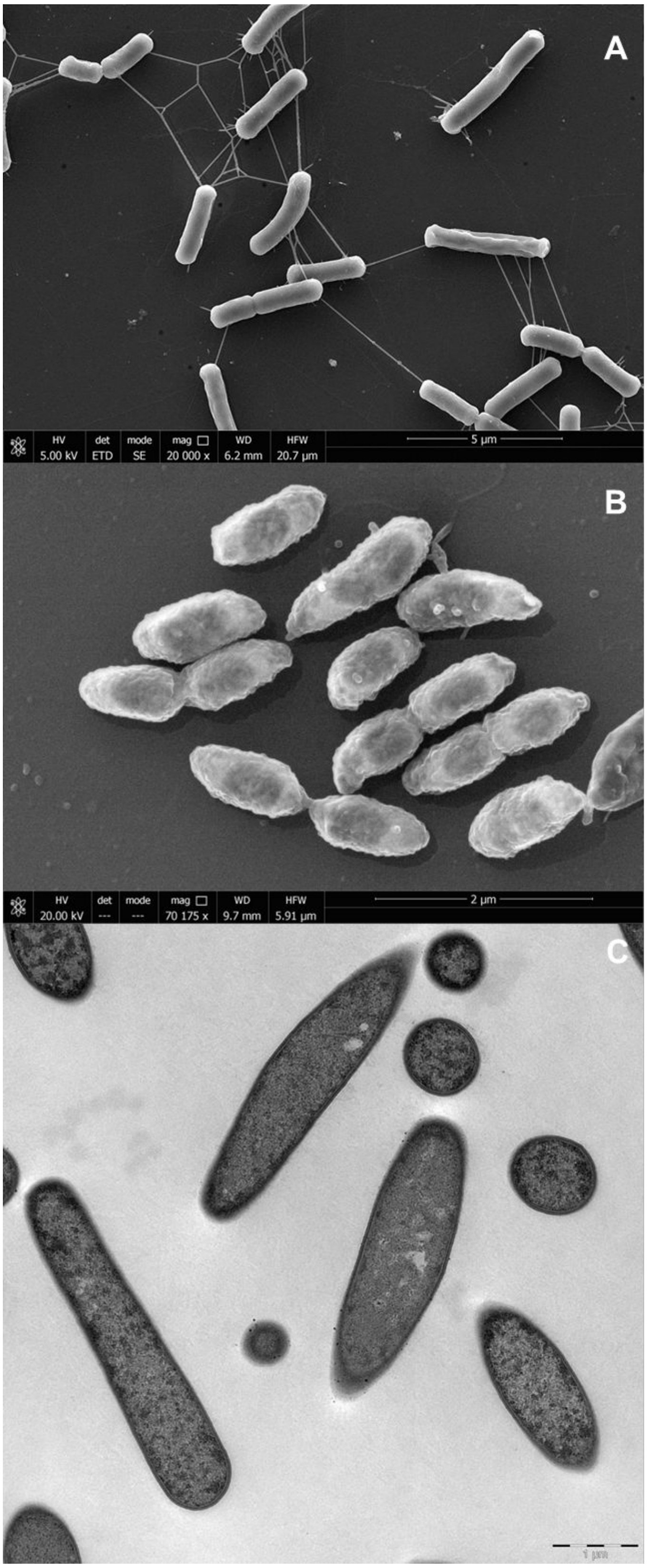

Figure S8. Environmental scanning electron microscopy images of untreated *L. sphaericus* (A, secondary electrons only) and *S. bentonitica* (B, merge of secondary and backscattered electrons). In C, a TEM micrograph of thin sections of untreated *L. sphaericus* is shown. Scale bars: 5 µm (A), 2 µm (B), and 1 µm (C).

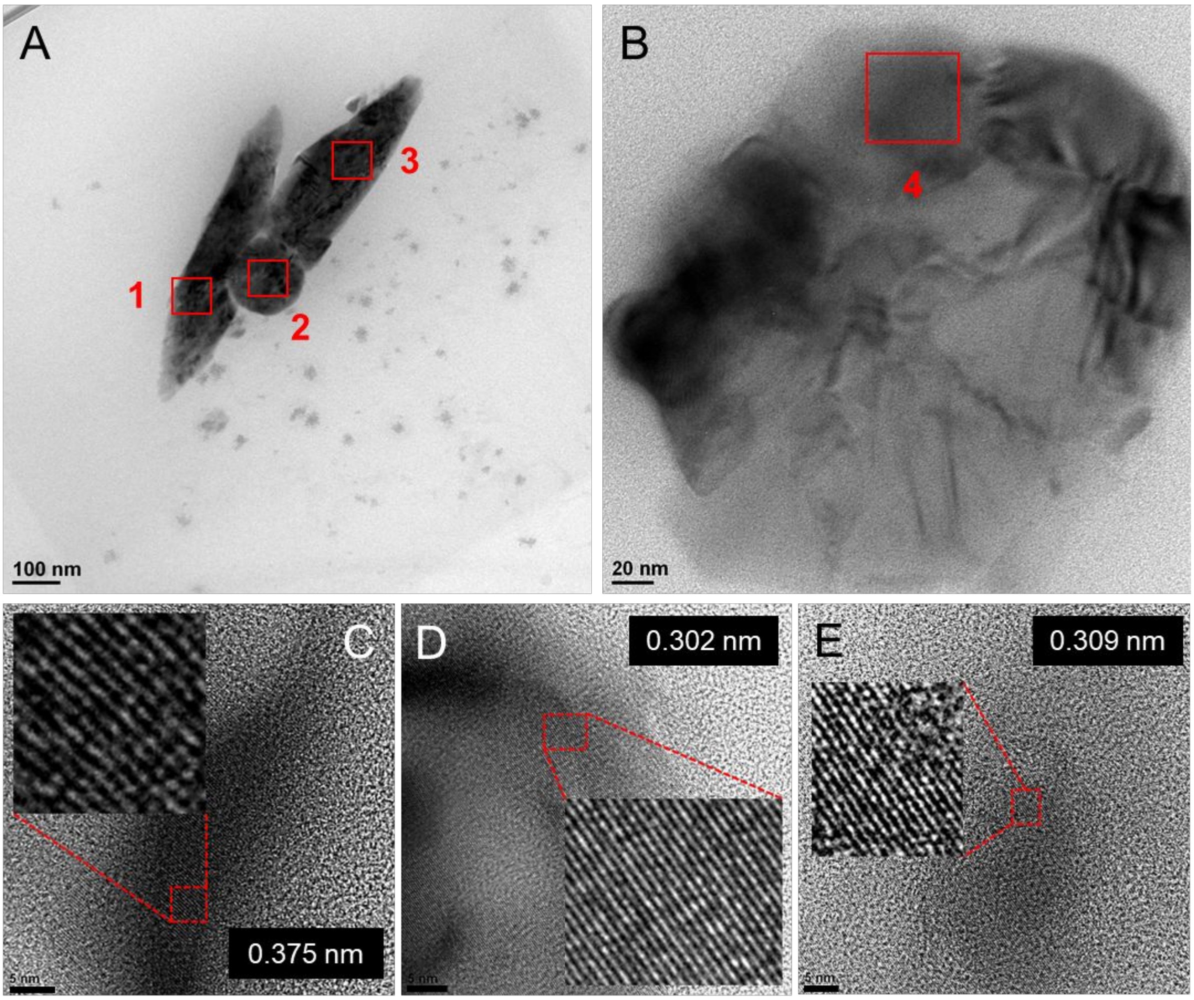

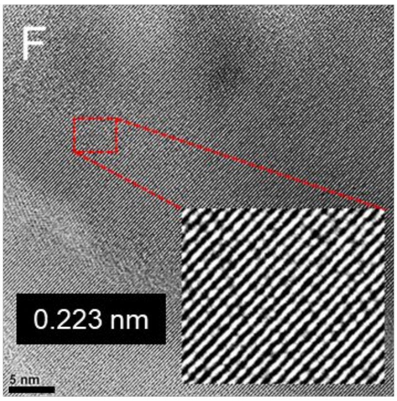

	Shape	Lattice spacing (nm)
**1 (C)**	polygonal	0.375
**2 (D)**	spherical	0.302
**3 (E)**	polygonal	0.309
**4 (F)**	hexagonal-like	0.223

Figure S9. Electron micrographs showing selenium nano- and microstructures (A, B) after exposure of *L. sphaericus* to 100 µM CS-SeNPs for 24 h. HR-TEM images (C-F corresponding to regions 1-4, respectively). The table summarizes measured lattice spacings. Scale bars: 100 nm (A), 20 nm (B), 5 nm (C-F).

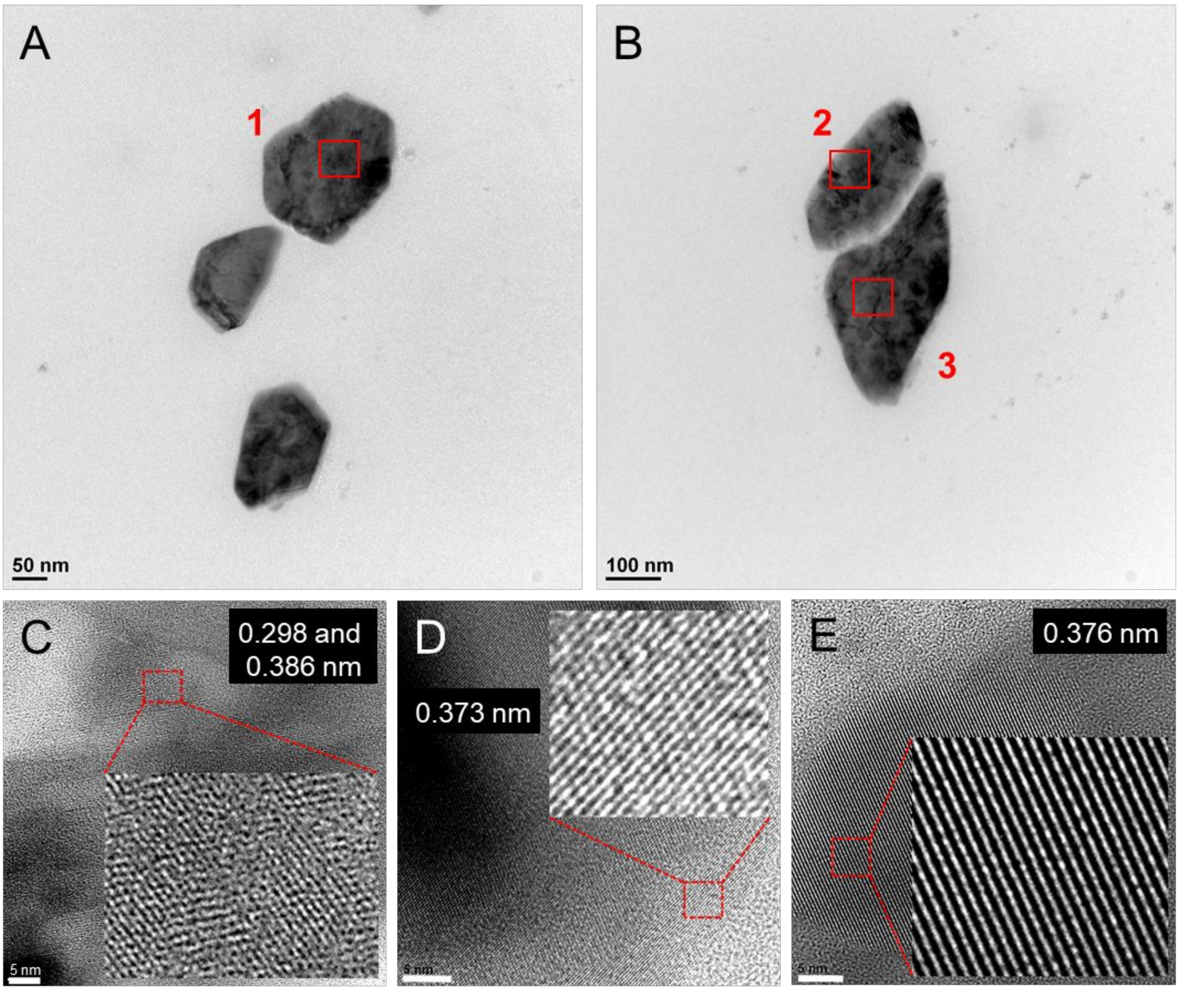

	Shape	Lattice spacing (nm)
**1 (C)**	hexagonal-like	0.298<br>0.386
**2 (D)**	polygonal	0.373
**3 (E)**	polygonal	0.376

Figure S10. Electron micrographs showing selenium nano- and microstructures (A, B) after exposure of *L. sphaericus* to 100 µM BSA-SeNPs for 24 h. HR-TEM images (C-E corresponding to regions 1-3, respectively). The table summarizes measured lattice spacings. Scale bars: 50 nm (A), 100 nm (B), 5 nm (C-E).

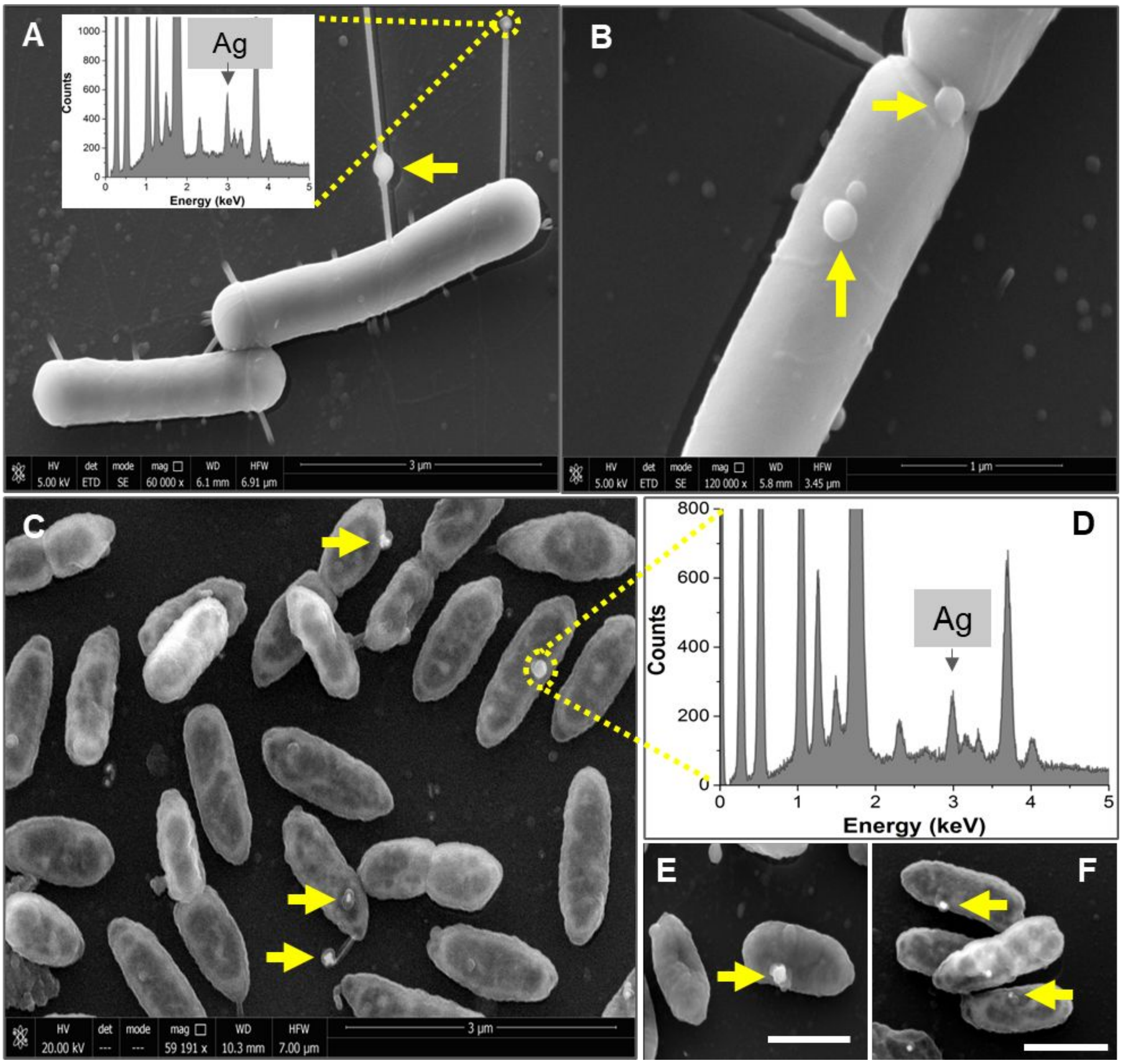

Figure S11. Environmental scanning electron microscopy images of *L. sphaericus* (A-B) and *S. bentonitica* (C-F) incubated for 24 h with 100 µM AgNPs (highlighted by arrows). Elemental composition of the NPs was investigated by EDX (panel in A, D). Scale bars: A (3µm), B (1µm) C (3µm) E (1 µm) and F (1µm).
